# Tailor‐Made Nanomaterials for Diagnosis and Therapy of Pancreatic Ductal Adenocarcinoma

**DOI:** 10.1002/advs.202002545

**Published:** 2021-02-12

**Authors:** Xi Hu, Fan Xia, Jiyoung Lee, Fangyuan Li, Xiaoyang Lu, Xiaozhen Zhuo, Guangjun Nie, Daishun Ling

**Affiliations:** ^1^ Department of Clinical Pharmacy Zhejiang Provincial Key Laboratory for Drug Evaluation and Clinical Research the First Affiliated Hospital Zhejiang University School of Medicine Hangzhou 310003 China; ^2^ Institute of Pharmaceutics Zhejiang Province Key Laboratory of Anti‐Cancer Drug Research Hangzhou Institute of Innovative Medicine College of Pharmaceutical Sciences Zhejiang University Hangzhou 310058 China; ^3^ Key Laboratory of Biomedical Engineering of the Ministry of Education College of Biomedical Engineering & Instrument Science Zhejiang University Hangzhou 310058 China; ^4^ Department of Cardiology the First Affiliated Hospital Xi'an Jiaotong University Xi'an 710061 China; ^5^ CAS Key Laboratory for Biomedical Effects of Nanomaterials and Nanosafety CAS Center for Excellence in Nanoscience National Center for Nanoscience and Technology No.11 Zhongguancun Beiyitiao Beijing 100190 China; ^6^ GBA Research Innovation Institute for Nanotechnology Guangzhou 510700 China

**Keywords:** diagnosis, imaging‐guided therapy, nanomaterials, pancreatic ductal adenocarcinoma, therapy

## Abstract

Pancreatic ductal adenocarcinoma (PDAC) is one of the deadliest cancers worldwide due to its aggressiveness and the challenge to early diagnosis and treatment. In recent decades, nanomaterials have received increasing attention for diagnosis and therapy of PDAC. However, these designs are mainly focused on the macroscopic tumor therapeutic effect, while the crucial nano–bio interactions in the heterogeneous microenvironment of PDAC remain poorly understood. As a result, the majority of potent nanomedicines show limited performance in ameliorating PDAC in clinical translation. Therefore, exploiting the unique nature of the PDAC by detecting potential biomarkers together with a deep understanding of nano–bio interactions that occur in the tumor microenvironment is pivotal to the design of PDAC‐tailored effective nanomedicine. This review will introduce tailor‐made nanomaterials‐enabled laboratory tests and advanced noninvasive imaging technologies for early and accurate diagnosis of PDAC. Moreover, the fabrication of a myriad of tailor‐made nanomaterials for various PDAC therapeutic modalities will be reviewed. Furthermore, much preferred theranostic multifunctional nanomaterials for imaging‐guided therapies of PDAC will be elaborated. Lastly, the prospects of these nanomaterials in terms of clinical translation and potential breakthroughs will be briefly discussed.

## Graphical abstract

This review focuses on the cutting‐edge nanomedicines and nano–bio interactions in the heterogeneous microenvironment of pancreatic ductal adenocarcinoma (PDAC). Herein, this work provides a comprehensive overview of the designed fabrication of PDAC‐tailored functional nanomaterials for laboratory tests, medical imaging, therapy (e.g., chemotherapy, anti‐stromal therapy, radiotherapy, gene therapy, immunotherapy, and ablative therapy), and imaging‐guided therapy of PDAC.

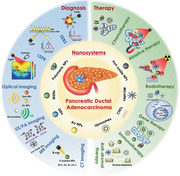

## 1 Introduction

### 1.1 Current Status and Challenges of PDAC Diagnosis and Treatment

Pancreatic ductal adenocarcinoma (PDAC) that accounts for more than 90% of all pancreatic cancers, is one of the most lethal cancers worldwide with an overall five‐year survival rate of <9%.^[^ ^1^ ^]^ The most common symptoms, including pain, jaundice, and weight loss, are subtle at onset and often lead to a delay in diagnosis. Unfortunately, only 15–20% of patients with diagnosed early‐stage PDAC can be eligible for surgical resection. Although surgical resection increases the five‐year survival rate to 15–25%, curative surgery alone is still inadequate as the recurrence rate for resected cases is as high as 85%.^[^ ^2^ ^]^

Thus, the validated laboratory tests and medical imaging examinations are essential in the diagnosis, surveillance, and follow‐up of the patients, despite of limited sensitivity and specificity. Regarding laboratory tests, the highly preferred cancer biomarker, serum carbohydrate antigen (CA) 19‐9, has relatively low sensitivity and specificity towards PDAC, and thus cannot be solely used as a criteria for the diagnosis of PDAC.^[^ ^3^ ^]^ Besides, the common clinical imaging modalities for PDAC diagnosis include computed tomography (CT), magnetic resonance (MR), and endoscopic ultrasound (EUS). Among them, CT is considered as the gold standard due to the outstanding sensitivity and accuracy, while having modest specificity.^[^ ^4^, ^5^ ^]^ Furthermore, CT or MR imaging can only detect the tumors with a diameter over 1 cm.^[^ ^6^ ^]^ EUS can detect PDAC as small as 2–3 mm, whereas is not readily accessible and mainly dependent on the skill of the operator. Therefore, current diagnostic technologies fail to offer satisfying precision for the critical diagnosis of PDAC or subserve patient stratification for an optimal treatment modality, such as surgery or systemic (neoadjuvant) therapy.

Over the past few decades, rigorous efforts towards various therapeutic modalities, for instance, chemotherapy, radiotherapy, immunotherapy, anti‐stromal therapy, and so on, have been made to ameliorate the PDAC treatment.^[^ ^6^, ^7^ ^]^ Under metastatic conditions, modern combinations of cytotoxic therapies including FOLFIRINOX/modified FOLFIRINOX (oxaliplatin, irinotecan, leucovorin, and 5‐fluorouracil (5‐FU)) and gemcitabine (GEM) + albumin‐bound paclitaxel (nab‐paclitaxel) are standard first‐line regimens for neoadjuvant therapy or patients with locally advanced PDAC.^[^ ^7^ ^]^ Unfortunately, thus far, limited success has been observed to extend the survival of PDAC patients.^[^ ^2^ ^]^ In general, PDAC is characterized by excessive fibrotic stroma, accounting for > 90% of the total tumor volume. The heterogeneous tumor microenvironment (TME) is composed of cancer‐associated fibroblasts (CAFs), pancreatic stellate cells (PSCs), regulatory T cells, tumor‐associated macrophages (TAMs), and a mass of extracellular matrix (ECM).^[^ ^8^ ^]^ On the one hand, PDAC stroma has increased the interstitial fluid pressure and reduced microvascular density, thus preventing drugs from penetrating the tissue interstitium.^[^ ^9^ ^]^ On the other hand, the fibrotic stroma is involved in the recruitment and activation of CAFs, extensive ECM deposition and remodeling, and the suppression of antitumor immune response.^[^ ^8^ ^]^ More importantly, the interaction between tumor cells and TME components favors the undesirable rapid progression, invasion and metastasis of PDAC.^[^ ^10^ ^]^ Concurrently, the high interstitial fluid pressure, hypoxic condition, and acidic extracellular pH in TME are believed to tragically promote tumorigenesis and tumor progression.^[^ ^11^ ^]^

### 1.2 Nanotechnology Brings a New Hope for PDAC

As suggested from numerous studies, nanomedicines have the immense potential to enhance the clinical outcomes in cancer patients, for example, clinical‐stage nanomedicines including Abraxane (nab‐paclitaxel),^[^ ^12^ ^]^ Doxil (liposomal doxorubicin),^[^ ^13^ ^]^ Onivyde (liposomal irinotecan),^[^ ^14^ ^]^ liposomal vincristine,^[^ ^15^ ^]^ MM 302 (HER2‐targeted liposomal doxorubicin),^[^ ^16^ ^]^ NanoTherm (iron oxide nanoparticles (NPs)),^[^ ^17^ ^]^ and so on. In addition to conventional drug delivery systems (e.g., micelles and liposomes), the nanomedicine communities are continuously developing novel functional nanomaterials, such as gold (Au) NPs, quantum dots (QDs), carbon nanotubes (CNTs), mesoporous silica NPs (MSNs), and so on, which are feasible to incorporate therapeutic agents and/or imaging probes.^[^ ^18^ ^]^ Nevertheless, the therapeutic efficacy of passive tumor targeting nanomedicine is not always satisfying because the well‐known enhanced permeability and retention (EPR) effect could be largely influenced by the thickness and density of the ECM, uneven blood flow distribution as well as the disproportionate vessel permeability in highly heterogeneous PDAC microenvironment.^[^ ^19^ ^]^ Moreover, the current designs of nanomaterials are mainly focused on the macroscopic tumor therapeutic effect, while the crucial nano–bio interactions in the heterogeneous microenvironment of PDAC remain poorly understood. Therefore, exploiting the unique nature of the PDAC via a deep understanding of nano–bio interactions that occur in the tumor microenvironment, is pivotal to the design of PDAC‐tailored effective nanomedicine.

All in all, it is crucial for researchers to focus on PDAC heterogeneous microenvironment and nano–bio interactions for overcoming the limitations of conventional EPR‐based nanomedicine, by: 1) utilizing TME‐specific molecular receptors (termed as “active targeting”) and biophysical properties (termed as “TME responsive”),^[^ ^20^ ^]^ 2) physiological remodeling (via anti‐stromal therapy) or physical alteration (via local ablative therapies) of TME.^[^ ^21^ ^]^ As to PDAC‐specific receptors, mesothelin, urokinase‐type plasminogen activator receptor (uPAR), cell‐surface associated mucin 1 (MUC1), epidermal growth factor receptor (EGFR)/Her‐2, plectin‐1 (PL‐1),^[^ ^22^ ^]^ insulin‐like growth factor 1 receptor (IGF1R), transferrin (Tf) receptor, and so on, have been reported to be overexpressed on the surface of the PDAC cells.^[^ ^23^ ^]^ Besides, the biophysical properties, for instance, low pH, redox, hypoxia, and enzymes (e.g., overexpressed matrix metalloproteinases (MMPs)) favor the development of stimulus‐responsive nanomedicines, which could be selectively triggered to magnify the therapeutic efficiency and/or imaging signals for PDAC treatment and/or diagnosis with on demand drug delivery and release.

### 1.3 Rational Design of Nanomaterials for the Diagnosis and Therapy of PDAC

**Figure 1:**
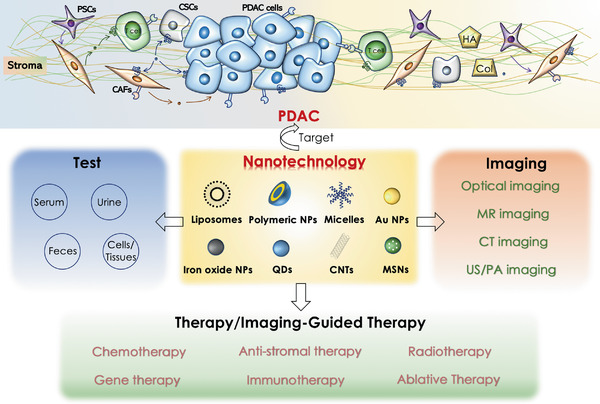
The development of tailor‐made nanomaterials in laboratory tests, medical imaging, therapy, and imaging‐guided therapy of heterogeneous PDAC. Liposomes, micelles, polymer NPs, and inorganic NPs can be easily modificated or loaded with therapeutic/imaging agents. Au NPs, QDs, and CNTs with intrinsic optical properties can be used for biosensing and biological imaging. Moreover, iron oxide NPs and high‐Z element NPs (e.g., Au NPs) can be utilized for MR imaging and CT imaging, respectively.

This review will focus on the cutting‐edge nanotechnology in view of PDAC heterogeneous microenvironment and provide a comprehensive overview of the rational fabrication of PDAC‐tailored nanomaterials (e.g., liposomes, micelles/polymeric nanomaterials, as well as protein‐based, inorganic and hybrid nanomaterials), which is beneficial for the development of diagnosis, therapy, and imaging‐guided therapy of PDAC (**Figure** **1**). We will summarize PDAC‐tailored nanomaterials for biochemical and immunological‐based laboratory tests of PDAC biomarkers (e.g., proteins, nucleic acids, extracellular vesicles, etc.), and advanced noninvasive imaging modalities. Furthermore, we will elaborate the active agents, versatile drug delivery nanosystems and other functional nanomaterials that mediate various therapeutic modalities (e.g., chemotherapy, anti‐stromal therapy, radiotherapy, gene therapy, immunotherapy, and ablative therapy, etc.) via targeting and/or modulating the unique components/characteristics in PDAC heterogeneous microenvironment to solve the therapeutic and theranostic dilemma of PDAC. Finally, the prospects of these PDAC‐tailored nanotechnologies in terms of clinical translation and potential breakthroughs will be briefly discussed.

## 2 PDAC‐Tailored Nanomaterials for Diagnosis

**Figure 2:**
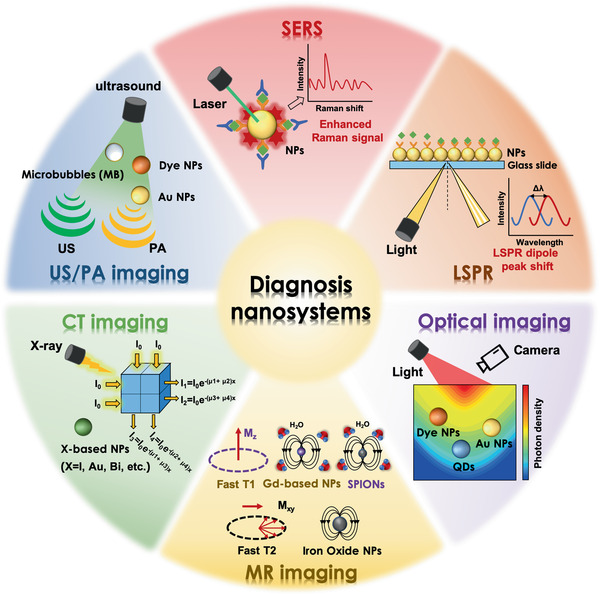
Illustration for various nanomaterials in the diagnosis of PDAC, including tailor‐made nanomaterials‐based in vitro detection technologies (e.g., SERS, LSPR) and various nanomaterials‐enabled in vivo imaging modalities (e.g., optical imaging, MR imaging, CT imaging, and US/PA imaging).

Due to the rare symptoms and limited diagnosis of PDAC, most of the patients have locally advanced or distant metastases upon diagnosis, and thus only 15–20% of patients are suitable for surgical resection after the initial diagnosis.^[^ ^3^ ^]^ Therefore, there is an urgent need for much more efficient laboratory tests and imaging technologies to achieve the diagnosis of PDAC. This part would introduce the development of tailor‐made nanomaterials for in vitro laboratory detection of biomarkers (e.g., proteins, nucleic acids, metabolites, etc.) as well as the nanomaterial‐enabled imaging modalities (e.g., MR imaging, CT imaging, US/PA imaging, and optical imaging, etc.) to improve the diagnosis of PDAC (**Figure** **2**).

### 2.1 PDAC‐Tailored Nanomaterials for Laboratory Tests

In this section, we will introduce the fabrication and efficiency of tailor‐made nanomaterials for laboratory detection of biomarkers (e.g., proteins, nucleic acids, extracellular vesicles, etc.) from the samples of blood, urine, or cells/tissues for PDAC diagnosis and prognosis (**Table** **1**).

**Table 1:** Summary of studies on the PDAC‐tailored nanomaterials for laboratory tests

Biomarker	Nanomaterials	Sample	Sources	Detection limit	Ref
CA19‐9	AuNPs@PThi	Serum	Human	0.26 U mL^−1^	^[^ ^29^ ^]^
Carcinoembryonic antigen (CEA)	CEA detection antibody‐AuNPs	Serum	Human	12 ng L^−1^	^[^ ^30^ ^]^
Mucin protein MUC4	AuNPs functionalized with Raman reporter molecules and specific antibodies	Serum	Human	<62.5 ng L^−1^	^[^ ^31^ ^]^
miR‐21, miR‐10b	Au nanoprisms functionalized with HS‐C6‐ssDNA	Plasma	Human	miR‐21: 23–35 × 10^−15^ m miR‐10b: 100 × 10^−9^ m‐50 × 10^−15^ m	^[^ ^35^ ^]^
miR‐10b, miR‐10a	Au nanoprisms functionalized with complementary oligonucleotides	Plasma	Human	miR‐10b: 83 × 10^−18^ m miR‐10a: 75 × 10^−18^ m	^[^ ^36^ ^]^
EV EpCAM, EV EphA2	Antibody‐conjugated QDs	Serum	Human	EV EpCAM: 1.9 × 10^8^ EVs EV EphA2: 2 × 10^8^ EVs	^[^ ^39^ ^]^
Glypican‐1, CD63	Alternating current electrokinetic (ACE) microarray chip	Whole blood, plasma, or serum	Human	–	^[^ ^40^ ^]^
Panc‐1‐derived exosomes	Polydopamine (PDA)‐modified immunocapture substrates and ultrathin PDA‐encapsulated antibody‐reporter‐Au@Ag multilayer (PEARL) nanotags	Serum	Human	9 × 10^−19^ mol L^−1^	^[^ ^41^ ^]^
REG1A	Nano‐biotinylated liposome‐based immuno‐loop‐mediated isothermal amplification	Urine	Human	1 fg mL^−1^	^[^ ^43^ ^]^
CA19‐9	SPIDE/CNO‐GO‐Ab	Cell lysates of colorectal adenocarcinoma	HT‐29 cells, SW‐620 cells	0.12 U mL^−1^	^[^ ^44^ ^]^
CEA	Aluminum‐based quantum structure (QS)	Cells	ASPC‐1 cells, H69 cells	–	^[^ ^45^ ^]^
Claudin‐4	CdSe(ZnS) QDs conjugated with modified apoferritin and anticlaudin 4	Cells	Capan‐1 cells	–	^[^ ^46^ ^]^
Claudin‐4 and prostate stem cell antigen (PSCA)	Indium phosphide@zinc sulfide (InP/ZnS) QDs conjugated with anticlaudin 4 and antiprostate stem cell antigen	Cells	Miapaca cells, Panc‐1 cells	–	^[^ ^47^ ^]^
F19 antigen	AuNPs conjugated with F19 monoclonal antibodies	Tissue samples	Human	–	^[^ ^6^ ^]^

#### 2.1.1 Detection of Protein‐Based Biomarkers in Blood

Presently, CA19‐9, a tumor‐associated glycoprotein, is the most extensively used biomarker for the clinical diagnosis of PDAC. The CA19‐9 levels of normal adults are generally lower than 37 U mL^−1^, and its slight elevation in the blood is closely related to PDAC incidence and development.^[^ ^24^ ^]^ However, CA19‐9 may also be significantly up‐regulated in patients with biliary infection or obstructive, which may induce the false‐positive results of PDAC diagnosis. Besides, in Lewis antigen‐negative individuals, CA19‐9 may be undetectable, resulting in false‐negative results.^[^ ^25^ ^]^ Thus, CA19‐9 blood test is only recommended for monitoring progress and treatment response, rather than for screening or diagnosis of PDAC. Therefore, enhancing the sensitivity of CA19‐9 test or exploring new biomarkers of PDAC is becoming the main focus.^[^ ^26^ ^]^

In recent years, a lot of attention has been focused on promoting the sensitivity of CA19‐9 probes for the early clinical diagnosis of PDAC. A wide variety of testing techniques zoom on the horizon for the detection of cancer biomarkers, predominantly utilizing electrochemical^[^ ^27^ ^]^ or optical transducers.^[^ ^28^ ^]^ The electrochemical transducer translates the interaction of the biomarker and biorecognition molecules into measurable electrochemical signals, such as current, potential, conductance, and impedance. Huang et al. developed polythionine‐Au composites (AuNPs@ PThi)‐ and anti‐CA19‐9 antibodies‐immobilized glassy carbon electrode as a sensitive redox probe for label‐free electrochemical immunoassay. The fabricated immunosensor achieved ultrasensitive detection of CA19‐9 in a linear range from 6.5 to 520 U mL^−1^, and the detection limit was 0.26 U mL^−1^ at a signal‐to‐noise ratio of 3. The accuracy and convenience were tested using clinical serum samples of PDAC and normal control.^[^ ^29^ ^]^ Therefore, such tailor‐made nanomaterials for biochemical and immunological strategies have the potential to overcome the hurdles during laboratory tests of the PDAC by enhancing the sensitivity and specificity of CA19‐9 test.

Besides CA19‐9, Au NPs‐ and QDs‐based optical nanoprobes for other cancer biomarkers, for instance, carcinoembryonic antigen (CEA)^[^ ^30^ ^]^ and mucin protein MUC4^[^ ^31^ ^]^ have also been developed for PDAC diagnosis, ranging from immunoassays to live cell/tissue imaging.^[^ ^32^ ^]^ For example, Liu et al. reported an enzyme‐labeled Au nanoprobe by coating AuNP with antibody, single‐stranded DNA (ssDNA), and horseradish peroxidase (HRP, as signal amplification). The enzyme‐labeled Au nanoprobes achieved a detection limit of 12 ng L^−1^ for CEA in human serum samples, whose sensitivity was about 130 times higher than that of conventional enzyme‐linked immunosorbent assay (ELISA).^[^ ^30^ ^]^ As to mucin protein MUC4 as an overexpressed protein, Krasnoslobodtsev et al. functionalized Au NPs with Raman reporter molecules (RRM's) and specific antibodies for Surface Enhanced Raman Scattering (SERS)‐based nano‐immunoassay, which successfully detected low levels of mucin protein MUC4 in PDAC patients serum.^[^ ^31^ ^]^

#### 2.1.2 Detection of Nucleic Acid‐Based Biomarkers in Blood

**Figure 3:**
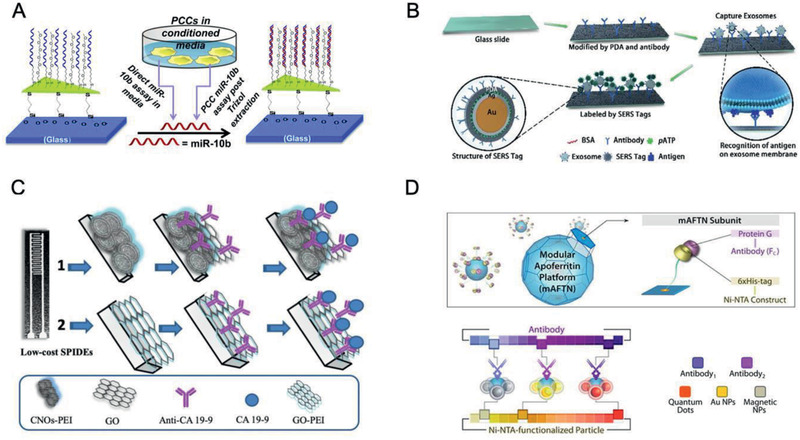
PDAC‐tailored nanomaterials for laboratory tests. A) Schematic illustration of the fabrication of the glass substrate‐bound Au nanoprisms to prepare LSPR‐based microRNA sensor for miR‐10b detection in plasma samples.Reproduced with permission.^[^ ^36^ ^]^ Copyright 2015, American Chemical Society. B) Schematic illustration of the construction of chip‐based exosome‐PEARL SERS immunosensor. Reproduced with permission.^[^ ^41^ ^]^ Copyright 2018, Royal Society of Chemistry. C) Illustration of fabrication of 1) SPIDE/CNO‐GO‐Ab and 2) SPIDE/GO‐Ab and CA19‐9 detection process. Reproduced with permission.^[^ ^44^ ^]^ Copyright 2019, Elsevier. D) Apoferritin‐based modular nanoplatform (e.g., QDs, Au, or magnetic nanoprobes) for the detection of claudin 4. Reproduced with permission.^[^ ^46^ ^]^ Copyright 2013, American Chemical Society.

In addition to the standard cancer biomarkers, nucleic acid‐based biomarkers in body fluids of patients are of great importance to detect early cancers at which no symptoms are present. Among them, miRNAs that often play a major role in cell proliferation, invasion, and metastasis in various cancers (including PDAC), are the nucleic acid‐based biomarkers for cancer diagnosis.^[^ ^33^ ^]^ It has been reported that miR‐10b, miR‐21, miR30c, miR‐132, miR‐155, and miR‐212 are markedly overexpressed in PDAC cells compared with nonmalignant cells, which is conducive to the differentiation of plasma levels between PDAC patients and patients without pancreatic pathology.^[^ ^34^ ^]^ Sardar and Korc group designed a solid‐state localized surface plasmon resonance (LSPR) sensor to detect the sub‐femtomolar concentration of miR‐21 and miR‐10b in human plasma for PDAC patients. In this plasmonic biosensor, Au nanoprisms functionalized by complementary single‐stranded DNAs (HS‐C6ssDNA) were attached to a glass substrate, and the changes in *λ*_LSPR_ of the Au nanoprisms reflected the concentration of multiple miRs.^[^ ^35^ ^]^ They further applied the biosensor to distinguish between miR‐10b and miR‐10a without RNA extraction, and readily differentiate miR‐10b levels between PDAC and chronic pancreatitis patients (**Figure** **3**A).^[^ ^36^ ^]^

Moreover, Xu et al. reported an *α*‐hemolysin (*α*HL) single nanochannel which was constructed via self‐assembly of *α*HL and a phospholipid bilayer at a +180 mV transmembrane voltage. The *α*HL single nanochannel successfully detected the miR‐21 signal and effectively differentiated the complex molecules signal of miR‐21·probe 21, miR‐155·probe 155, and miR‐196a·probe 196a.^[^ ^37^ ^]^ Thus, considering the specificity of nucleic acid‐based biomarkers, the tailor‐made nanomaterials for the laboratory test provide a perfect adjuvant method for PDAC early detection.

#### 2.1.3 Detection of Vesicles‐Based Biomarkers in Blood

As another potential biomarker for cancer detection, extracellular vesicles (EVs, subdivided into exosomes and microvesicles) are abundantly secreted by cancer cells, and carry biomarkers that reflect the phenotype of their parental cells.^[^ ^38^ ^]^ Rodrigues et al. fabricated a rapid NPs‐ and dye based fluorescent immunoassay, in which EVs from the serum were stained with a lipophilic dye DiO (3,3’‐dihexadecyloxacarbocyanine perchlorate) and then hybridized with antibody‐conjugated QDs probes to detect specific biomarkers. The probe‐to‐dye signal ratio could determine the mean expression of PDAC‐associated EV membrane biomarkers, for example, ephrin type‐A receptor 2 (EphA2) and epithelial cell adhesion molecule (EpCAM), and thereby differentiate patients with and without PDAC.^[^ ^39^ ^]^ Moreover, Lewis et al. reported on an alternating current electrokinetic (ACE) microarray chip device that can isolate exosome and other EVs from 25 µL of undiluted blood, plasma, or serum sample of PDAC patients in 20 min. Thereafter, on‐chip immunofluorescence analysis of the biomarkers glypican‐1 (GPC‐1) and CD63 (exosome‐associated biomarker) can be performed directly. The results exhibited that PDAC patient samples (*n* = 20) were distinguished from healthy samples (*n* = 11) with 99% sensitivity and 82% specificity.^[^ ^40^ ^]^

Furthermore, exosomes can reveal PDAC cell information, such as the metabolic state and degree of histological malignancy. Li et al. developed polydopamine (PDA)‐modified glass substrates and ultrathin PDA‐encapsulated antibody‐reporter‐Au@Ag multilayer (PEARL) nanotags with Raman reporters at 1072 cm^−1^. The chip‐based exosome‐PEARL SERS immunosensor captured the exosomes originated from PDAC serum samples (2 µL of volume), which can be recognized by PEARL SERS nanotags to form a “chip‐exosome‐PEARL tag” sandwich structure for Raman spectrum scanning. The migration inhibitory factor (MIF) antibody‐based SERS immunoassay discriminated PDAC patients (*n* = 71) from healthy ones (*n* = 32). More surprisingly, it distinguished metastasized tumors from the nonmetastatic tumors, as well as tumor node metastasis (TNM) of P1‐2 stages from the P3 stage. The novel immunoassay is time‐saving and does not require high‐speed ultracentrifugation processes; therefore, it could achieve ultrasensitive and specific detection, classification and metastasis monitoring of PDAC patients (Figure 3B).^[^ ^41^ ^]^

#### 2.1.4 Detection of Biomarkers in Urine

Furthermore, urine is one of the most attractive biofluids in clinical practice because it could be obtained in a noninvasive manner and could be stored easier than other body fluids. Liu et al. reported a nano‐biotinylated liposome‐based immuno‐loop‐mediated isothermal amplification (LAMP) for the ultrasensitive and specific detection of REG1A protein, which is a biomarker for PDAC in the urine. LAMP is a great genetic amplification procedure, which employs four primers that identify six or four regions on the target DNA, thus possessing superior specificity and sensitivity than PCR.^[^ ^42^ ^]^ The detection range of REG1A was from 1 fg mL^−1^ to 1 µg mL^−1^, and the detection limit was 1 fg mL^−1^. REG1A concentrations in patient samples detected by the LI‐LAMP assay were in compliance with those by ELISA.^[^ ^43^ ^]^

#### 2.1.5 Detection of Protein‐Based Biomarkers of PDAC Cells/Tissues

The specific optical nanoprobes are also served as promising candidates for the early detection of human PDAC cells, extending the in vitro cancer diagnosis. For example, Ibáñez‐Redín et al. modified Ag screen‐printed interdigitated electrodes (SPIDEs) by using carbon nano‐onions (CNOs) and graphene oxide (GO) films immobilized with anti‐CA19‐9 antibodies. SPIDE/CNO‐GO‐Ab biosensor provided a highly sensitive detection of CA19‐9 with a relatively low detection limit of 0.12 U mL^−1^ (Figure 3C).^[^ ^44^ ^]^ Also, Ganesan et al. presented an unique 3D biocompatible aluminum‐based quantum structure (QS) for CEA via SERS, which could differentiate PDAC cells and normal cells by intensity contrast.^[^ ^45^ ^]^ Besides, claudin 4 as an integral membrane PDAC biomarker has been detected using nanoprobes. Hwang et al. functionalized CdSe (ZnS) QDs and Au NPs with nickel‐nitriolotriacetic acid (Ni‐NTA) and conjugated them with modified apoferritin (mAFTN) and claudin 4 antibodies. The fluorescence detection sensitivity of mAFTN‐QDs nanoprobes to claudin 4 was 27‐fold, which is sixfold higher than that of conventional organic fluorophores and single QDs (Figure 3D).^[^ ^46^ ^]^ Moreover, Yong et al. reported that indium phosphide@zinc sulfide (InP/ZnS) QDs conjugated with anti‐claudin 4 and antiprostate stem cell antigen (anti‐PSCA). Strong uptake of anti‐PSCA‐conjugated InP/ZnS QDs and anti‐claudin 4‐conjugated InP/ZnS QDs was observed in Miapaca cells and low passage PDAC cell line XPA3, instead of receptor‐negative KB cells.^[^ ^47^ ^]^

To improve the diagnostic accuracy of PDAC, Eck et al. developed mAb‐F19‐conjugated Au nanoprobes for facile identification of PDAC tissues, in which mAb‐F19 recognizes the FAP abundantly expressed by reactive stromal fibroblasts of PDAC. mAb‐F19‐conjugated Au nanoprobes could label tumor stroma in ≈5 µm thick sections of resected human PDAC tissue, which were imaged by darkfield microscopy at ≈560 nm. The darkfield microscopy images displayed produced signals in PDAC tissues rather than in noncancerous pancreatic tissues, potentially allowing for in vivo laparoscopic imaging.^[^ ^6^ ^]^

Overall, more potential biomarkers are needed to be identified in PDAC, and nanotechnology‐based detection strategies are extremely important to improve the sensitivity, specificity, and overall accuracy of PDAC diagnosis.

### 2.2 PDAC‐Tailored Nanomaterials for Medical Imaging

Besides laboratory detection of biomarkers, clinical imaging protocols have been also employed to detect the primary tumor and distant metastasis, determine the resectability, evaluate treatment response. Moreover, real‐time therapeutic surveillance is gradually worthy of wide attention to timely evaluate the success of a treatment regimen and detect recurrence.^[^ ^48^ ^]^ Numerous imaging techniques including CT, MRI, and EUS, play important roles in the early diagnosis of PDAC. However, only tumors with a diameter of over 1 cm can be detected by current clinical imaging protocols including CT or MR imaging.^[^ ^6^ ^]^ Particularly, diagnostic results of EUS are largely dependent on the experience of operators and the body condition of patients.^[^ ^4^ ^]^ Therefore, strategies to increase the sensitivity and specificity of PDAC imaging remains as a formidable challenge. Herein, the efforts toward improving tailor‐made diagnostic nanomaterials that are beneficial for PDAC imaging modalities, would be introduced as follows (Figure 2; **Table** **2**).

**Table 2:** Summary of studies on the PDAC‐tailored nanomaterials for medical imaging

Nanosystems	Targeting ligands	Imaging Components	Imaging	Source	Cell Lines	In vivo model	Outcome	Ref
bMSN@Cy7.5‐FA NPs	Folic acid (FA)	Cy7.5	Optical imaging	IVIS system with *λ*_ex_ 740 nm and *λ*_em_ 790 nm	BxPC‐3 cells	BxPC‐3 orthotopic tumor model	Increase probe uptake rate in tumor tissues, in vivo fluorescence imaging of tumor metastasis	^[^ ^57^ ^]^
AP1153‐ICG‐CPSNPs	AP1153	ICG	Optical imaging	FX whole animal imager with *λ*_ex_ 755 nm and *λ*_em_ 830 nm	Panc‐1 cells	Panc‐1 orthotopic tumor model	Facilitate delivery of NPs to PDAC tumors in vivo, early detection of PDAC lesions	^[^ ^59^ ^]^
HSA‐GEM/IR780 nanocomplexes	HSA	IR780	Optical imaging	Maestro with 745 nm/785 nm filter setting	BxPC‐3 cells	BxPC‐3 subcutaneous tumor model	NIR imaging and chemotherapy	^[^ ^60^ ^]^
ENO1‐Dexg‐PCL/SPIO nanoparticles	Enolase 1	Superparamagnetic iron oxide NPs	MR imaging	1.5‐T MRI scanner	CFPAC‐1 cells, Miapaca‐2 cells	CFPAC‐1 subcutaneous tumor model	Increase the detection efficiency of PDAC	^[^ ^70^ ^]^
CXCR4‐iron oxide NPs	CXCR4 monoclonal antibody	Iron oxide NPs	MR imaging	1.5‐T MRI scanner	AsPC‐1 cells, BxPC‐3 cells, CFPAC‐1 cells, Panc‐1 cells	–	Semiquantitatively assess the cellular CXCR4 expression levels	^[^ ^71^ ^]^
Paclitaxel‐loaded perfluoropentane (PFP) nanoemulsions	‐	PFP	US imaging	1‐MHz ultrasound	Miapaca‐2 cells	Miapaca‐2 subcutaneous tumor model	US imaging and therapy of PDAC	^[^ ^80^ ^]^
EGFR‐conjugated Ag nanoplates	EGFR	Ag nanoplates	PA imaging	Pulsed light in the range of wavelengths between 740 and 940 nm	L3.6pl cells, MPanc96 cells	L3.6pl orthotopic tumor model	US and PA imaging of orthotopic pancreatic tumor	^[^ ^84^ ^]^
[^64^Cu]KRAS‑IGF1	IGF1 peptide analog	^64^Cu	PET imaging	Mosaic small animal PET scanner	AsPC1 cells	AsPC1 subcutaneous tumor model	Strong contrast signal of PDAC in PET imaging	^[^ ^88^ ^]^
Antihuman CD326‐grafted UCNPs‐based micelles	Antihuman CD326 antibody	Gd, UCNPs	Optical imaging, MR imaging	Maestro with 980 nm/650 nm filter 7‐T MRI scanner	BxPc‐3 cells	BxPc‐3 subcutaneous tumor model	Dual‐mode MR/UCL imaging	^[^ ^91^ ^]^
Glypican‐1‐antibody‐conjugated Gd‐Au nanoclusters (NCs)	Glypican‐1 antibody	Gd‐Au NCs	Optical imaging, MR imaging	IVIS system with *λ*_ex_ 535 nm and *λ*_em_ 670 nm 3‐T MRI scanner	COLO‐357 cells	COLO‐357 subcutaneous mouse model	Dual‐modal fluorescence/MR imaging	^[^ ^92^ ^]^
[^111^In]DOTA_n_‐poly(diamidopropanoyl)^m^‐KRAS2 PNA‐D(Cys‐Ser‐Lys‐Cys) nanoparticles	IGF1	^111^In(III), Gd	PET imaging, MR imaging	Starcam (GE Medical) gamma camera	AsPC1 cells	AsPC1 xenograft tumor mice model	Selective imaging mRNA expression in PDAC	^[^ ^93^ ^]^
Dextran‐coated iron oxide NPs conjugated with Cy5.5 and uMUC1‐specific peptide	uMUC1‐specific peptide	Iron oxide NPs, Cy5.5	MR imaging, optical imaging	9.4‐T MRI scanner IVIS system with specific filters for the Cy5.5 dye	–	Transgenic murine model of pancreatic cancer (KCM triple transgenic mice)	Imaging cellular levels of the tumor antigen uMUC1	^[^ ^94^ ^]^
RITC and EPCAM antibody functionalized iron oxide NPs	EPCAM antibody	Iron oxide NPs, RITC	MR imaging, optical imaging	7‐T MRI scanner Leica DM2500 microscope equipped with a Leica DFC350 FX Camera	Panc‐1 cells	–	Cancer‐specific targeting and fluorescent/MR imaging of Panc‐1 cells	^[^ ^95^ ^]^
Plectin‐SPION‐Cy7	Plectin‐1 antibody	Iron oxide NPs, Cy7	MR imaging, optical imaging	1.5‐T MRI scanner IVIS system with *λ*_ex_ 750 nm and *λ*_em_ 773 nm	Miapaca‐2 cells, XPA‐1 cells	XPA‐1 subcutaneous tumor model	High accumulation in the tumor mass instead of in normal tissues (e.g., pancreatic tissues, liver, or kidney)	^[^ ^96^ ^]^
Air‐filled polyvinyl alcohol microbubbles (PVA‐MBs) labeled with VivoTag‐680	–	Microbubbles, VivoTag‐680	MR imaging, optical imaging	IVIS system with *λ*_ex_ 675 nm and *λ*_em_ 720 nm Frequency 18 MHz US, power of 4%	KrasLSL‐G12D/+ cells, p53R172H/+ cells, Pdx1‐Cre (KPC cells)	Subcutaneous pancreatic tumor xenograft and orthotopic pancreatic tumor xenograft	Visualize blood flow within the tumor mass	^[^ ^97^ ^]^

#### 2.2.1 Optical Imaging

The clinical optical imaging that utilizes light with a wavelength ranging between 700 and 2000 nm has emerged as a powerful imaging method to provide real‐time feedback during surgeries.^[^ ^49^, ^50^, ^51^ ^]^ Near‐infrared (NIR) dyes, Au NPs, QDs, and rare‐earth doped NPs have received widespread interests in optical imaging of pancreatic tumors,^[^ ^52^ ^]^ among which IR780,^[^ ^53^ ^]^ IR820,^[^ ^54^ ^]^ cyanine (e.g., cy5.5,^[^ ^55^ ^]^ cy7,^[^ ^56^ ^]^ cy7.5,^[^ ^57^ ^]^ etc.) and indocyanine green (ICG) ^[^ ^58^ ^]^ are commonly applied NIR dyes.

**Figure 4:**
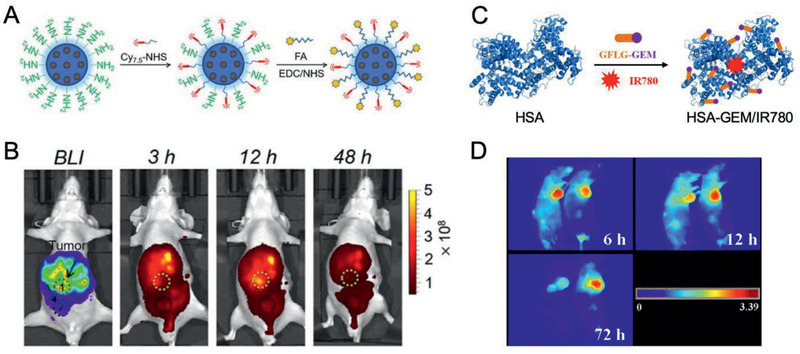
PDAC‐tailored nanomaterials for optical imaging. A) Synthesis of bMSN@Cy 7.5‐FA NPs. B) Fluorescence imaging of tumor metastasis sites after i.v. injection of bMSN@Cy 7.5‐FA NPs. Reproduced with permission.^[^ ^57^ ^]^ Copyright 2018, Elsevier. C) The construction of HSA‐GEM/IR780 nanocomplexes. D) Fluorescence imaging of nude mice bearing tumors after i.v. injection of HSA‐GEM/780 nanocomplexes. Reproduced with permission.^[^ ^60^ ^]^ Copyright 2017, Elsevier.

Li et al. prepared Cy 7.5‐ and FA‐conjugated biodegradable MSNs (bMSNs@Cy7.5‐FA NPs, ≈100 nm) to visualize tumors in vivo. The maximal fluorescence intensity in pancreatic metastatic tumors was observed at 12 h post‐injection. It demonstrated that bMSNs@Cy7.5‐FA NPs provided an excellent imaging platform for the early detection of tumor metastasis (**Figure** **4**A,B).^[^ ^57^ ^]^ Furthermore, Clawson et al. designed amorphous calcium phosphosilicate NPs (CPSNPs) that were doped with ICG and covalently coupled with CCKBR DNA aptamers 1153. The AP1153 could be taken up via a CCKBR (overexpressed in PDAC)‐mediated process without triggering CCKBR signaling or stimulating PDAC cell proliferation. After AP1153‐PEG‐ICG‐CPSNPs injection, tumor fluorescent reached a peak at 15–18 h in Panc‐1 orthotopic tumor models. Therefore, the AP‐targeted delivery system holds a promise for improved early diagnosis of PDAC lesions.^[^ ^59^ ^]^ Moreover, Han et al. constructed an HSA‐GEM/IR780 nanocomplex that showed better tumor retention ability than most cyanine NIR bioprobes. The strong fluorescent signals in PDAC were observed under NIR excitation even at 72 h post‐injection of HSA‐GEM/IR780 (Figure 4C,D).^[^ ^60^ ^]^

However, although NIR optical imaging is a powerful and noninvasive tool for PDAC imaging, many NIR dyes are the NIR‐I‐active with emission wavelength between 650 and 950 nm and have a limited penetration depth, which hinders their further applications in deep tumors. Thus, emission wavelength shifting to NIR‐II (1000–1700 nm) is desired due to the deeper penetration advantage of NIR‐II.^[^ ^51^ ^]^ Recently, Shao et al. for the first time reported chemically defined conjugated small molecules (CSMs) and their nanomedicines (CSMNs) with NIR‐II deep tissue penetration ability,^[^ ^61^ ^]^ which is a promising candidate for the optical imaging of PDAC.

#### 2.2.2 MR Imaging

MR imaging is widely used in clinical diagnosis with the superiority in providing additional details of tissue function and structure. Although MR imaging has sufficient sensitivity for imaging small sized cystic lesions, it has low sensitivity in the detection of solid lesions of PDAC.^[^ ^62^ ^]^ Moreover, gadolinium (Gd)‐based MR contrast agents have been applied in the early diagnosis of PDAC to enhance the sensitivity, however, their specificity of Gd‐enhanced MR imaging is still relatively low (63%).^[^ ^3^, ^63^ ^]^

In recent years, MR contrast agents have been widely studied for the early diagnosis of tumors. Generally, there are two types of contrast agents: T1‐weighted contrast agents, like Gd, MnO NPs, and extremely small iron oxide NPs (ESIONPs) (<4 nm),^[^ ^64^ ^]^ can reveal a bright signal enhancement and fine anatomic structures; while T2‐weighted contrast agents, such as superparamagnetic iron oxide NPs, generate intrinsic dark signals.^[^ ^65^ ^]^

Holbrook et al. functionalized spherical Au NPs (≈17.2 nm) with small molecule Gd (III) contrast agents (Lip‐Gd@AuNPs) to label and visualize the pancreas using T1‐weighted MR image. Significant contrast enhancement was observed for a clear identification of the pancreas with contrast‐to‐noise ratios above 35:1 after i.p. administration.^[^ ^66^ ^]^ However, Gd‐based contrast agents have potential toxicity, especially the risk of nephrogenic systemic fibrosis (NSF) in patients with advanced renal insufficiency.^[^ ^67^ ^]^

In recent decades, biocompatible iron oxide NPs have emerged as potential MR contrast agents and can avoid the toxicological risk of NSF.^[^ ^68^, ^69^ ^]^ Enolase 1 (ENO1, also named as pyruvate dehydrogenase 1), which is up‐regulated on cell membrane of PDAC, has been utilized for ENO1‐targeted imaging of PDAC. Wang et al. reported ENO1‐targeted superparamagnetic iron oxide NPs for specific MR molecular imaging of PDAC both in vitro (CFPAC‐1 and Miapaca‐2 cells) and in vivo (CFPAC‐1 subcutaneous tumor model).^[^ ^70^ ^]^ Moreover, He et al. focused on the important role of CXCR4 in the growth and metastasis of PDAC and reported CXCR4 mab‐modified iron oxide NPs for MR imaging of PDAC cell lines. The T2‐weighted MR enhancement and *Δ*r2 values of CXCR4‐iron oxide NPs semiquantitatively assessed the cellular CXCR4 expression levels in AsPC‐1, BxPC‐3, CFPAC‐1, and Panc‐1 cell lines. Therefore, CXCR4‐iron oxide NPs might be applied to predict prognosis with PDAC patients.^[^ ^71^ ^]^

However, the dark signals of lesions under T2‐weighted MR imaging may be confused with air, hemorrhage, blood clots, calcification and other hypointense areas.^[^ ^68^ ^]^ Thus, T1 contrast agents with bright signals are more desired for precise diagnosis. Currently, ESIONPs have been widely investigated for T1‐weighted MR imaging due to their facile large‐scale synthesis, high r1 values and low toxicity. And ESIONPs‐based TME responsive nanoassemblies, which are feasibly fabricated by using responsive polymers,^[^ ^72^ ^]^ cross‐linking,^[^ ^73^ ^]^ and i‐motif DNA,^[^ ^74^ ^]^ can achieve tumor‐specific T1‐weighted MR imaging amplification and serve as promising candidates for PDAC diagnosis. For example, the ESIONPs nanoassembly constructed by pH sensitive i‐motif DNAs could reveal the T2‐weighted dark MR signals in normal tissues and convert to T1‐weighted MR bright signals once the low pH of the TME triggers the disassembly into monodisperse ESIONPs. It can make tumor tissue exclusively bright among the dark normal tissue.^[^ ^74^ ^]^ This T2‐T1 inversion strategy offers a bright sight in the diagnosis of small‐sized tumor, thus provides a potential in the early diagnosis of PDAC and detection of metastasis lesions.

#### 2.2.3 CT Imaging

Contrast‐enhanced multi‐detector row CT is a routine diagnosis for suspicious PDAC lesions. However, the clinically used iodine‐based CT contrast agents show extremely short blood circulation half‐time, and thus the imaging detection results are largely rely on the contrast agents’ injection rate and the specific time after the injection.^[^ ^75^ ^]^ In addition, due to the low X‐ray attenuation coefficient of iodine, patients need to receive a large amount of iodine‐based agents, wherein the probability of adverse effects would further increase consequently.^[^ ^76^ ^]^ Therefore, CT contrast agents with higher X‐ray attenuation coefficient and greater biocompatibility are highly demanded for PDAC imaging.

Inorganic nanomaterials with remarkable X‐ray attenuation effects have been considered as potential candidates as CT contrast agents, including Au, bismuth (Bi), platinum (Pt), etc. Among them, Au NPs with a diameter of 20 nm have shown a high uptake rate in PDAC cells and thus are a promising candidate for CT imaging.^[^ ^77^ ^]^ Recently, tumor acidic microenvironment responsive bismuth subcarbonate nanotubes (BNTs) were reported for the tumor targeted CT imaging. Moreover, the large‐sized BNTs would disassemble into small‐sized bismuth subcarbonate clusters in tumor tissues, which can be easily cleared by the kidney and thus ensure safety.^[^ ^78^ ^]^ Currently, CT is a widely used imaging tool and a routine diagnostic modality for PDAC. Therefore, developing TME responsive nanomaterials is a promising way to enhance the specificity and sensitivity for the early diagnosis of PDAC.

#### 2.2.4 US/PA Imaging

Among many imaging modalities, US is cost effective and provides real‐time information, which is commonly used for the initial diagnosis of PDAC. However, the accuracy of US largely relies on the experience of operators and the condition of patients.^[^ ^4^ ^]^ Besides, as to tumors with poorly organized vessels or small lesions, contrast agents are needed to enhance the US imaging sensitivity.^[^ ^79^ ^]^ Furthermore, US‐mediated chemotherapy can be easily achieved by using drugs‐encapsulated polymeric micelles and/or nanoemulsions/microbubbles (MBs). For example, Rapoport et al. employed paclitaxel‐loaded perfluoropentane (PFP) nanoemulsions plus GEM in combination with a tumor‐directed 1‐MHz ultrasound. The US‐mediated PDAC therapy resulted in substantial regression of even large PDA tumors and dramatic suppression of metastases.^[^ ^80^ ^]^

Furthermore, laser‐induced PA imaging combines the superiorities of both optical and US imaging,^[^ ^81^ ^]^ and thus achieves great penetration depths (4–6 cm) and high resolution.^[^ ^82^ ^]^ PA imaging contrast agents, such as NIR dyes (e.g., ICG), CNTs, Au NPs, transition‐metal chalcogenides/MXene‐based NPs, etc. have been widely applied in tumor visualization.^[^ ^83^ ^]^ In addition, Ag nanoplates with the edge length of 128 ± 25.9 nm and thickness of 18 ± 2.7 nm showed the potential for PA imaging in orthotopic pancreatic tumor models.^[^ ^84^ ^]^ By virtue of the excellent optical properties of nanotechnology‐enabled PA contrast agents, the higher local contrast and greater penetration depth can be achieved. Therefore, tailor‐made nanomaterials for PA imaging show great potential in the diagnosis of PDAC, detection of tumor metastases and monitoring treatment responses,^[^ ^85^ ^]^ and moreover, their stability, potential toxicity, and sensitivity should be addressed.

#### 2.2.5 PET Imaging

In recent years, PET is an emerging diagnostic modality that has been successfully used in the diagnosis and monitoring of the prognosis of tumors due to its high sensitivity and specificity. Besides, it can provide auxiliary information for helping the decision‐making in advanced PDAC patients.^[^ ^86^ ^]^ However, some pressing issues remain to be solved. For example, ^18^F‐fluorodeoxyglucose‐based PET showed no obvious advantage in the early diagnosis of PDAC.^[^ ^86^, ^87^ ^]^ Besides, in patients with hyperglycemia or inflammatory masses, ^18^F‐fluorodeoxyglucose‐based PET may induce false‐negative or false‐positive results, due to the low glucose usage in these lesions.^[^ ^3^ ^]^ Nanotechnology‐enabled contrast agents can avoid such glucose metabolism dependent uptake of contrast agents.

Based on the KRAS oncogene, which activates in ≈95% of PDAC patients at the early pancreatic intraepithelial neoplasia (PanIN‐1) stage, Chakrabarti et al. designed a KRAS‑specific hybridization nanoprobe ([^64^Cu]KRAS‑IGF1) incorporating the positron‑emitting nuclide ^64^Cu and a cyclized IGF1 peptide analog. The [^64^Cu]KRAS‑IGF1 nanoprobe induced strong contrast signal in the center of human pancreas cancer xenografts by PET images, which was 8.6 ± 1.4‑fold higher than that in the contralateral muscle at 4 h post‑injection.^[^ ^88^ ^]^ In addition, Reiner and coworkers reported Doxil labeled with Zirconium‐89 nanoreporter (^89^Zr‐NRep) to precisely quantify the drug accumulation in tumor.^[^ ^89^ ^]^ Thus, tailor‐made nanomaterials for PET imaging have the potential to amplify the advantages of PET in PDAC diagnosis, especially in monitoring the therapeutic outcomes.

#### 2.2.6 Multimodal Imaging

Despite there are various imaging modalities for PDAC, all of these have their own advantages and inherent shortcomings. For example, PET/CT plus the endoscopic US may be helpful to the early diagnosis of PDAC due to the high sensitivity of PET/CT and the high specificity of endoscopic US.^[^ ^90^ ^]^ By performing two or more complementary imaging modalities without radio‐labeling, for instance, MR/optical imaging, US/optical imaging, and so on, multifunctional nanomaterials can synergistically offer more accurate imaging information, and thus contribute to the early diagnosis of the primary PDAC, evaluation for distant metastasis, and measurement of therapeutic outcomes.

**Figure 5:**
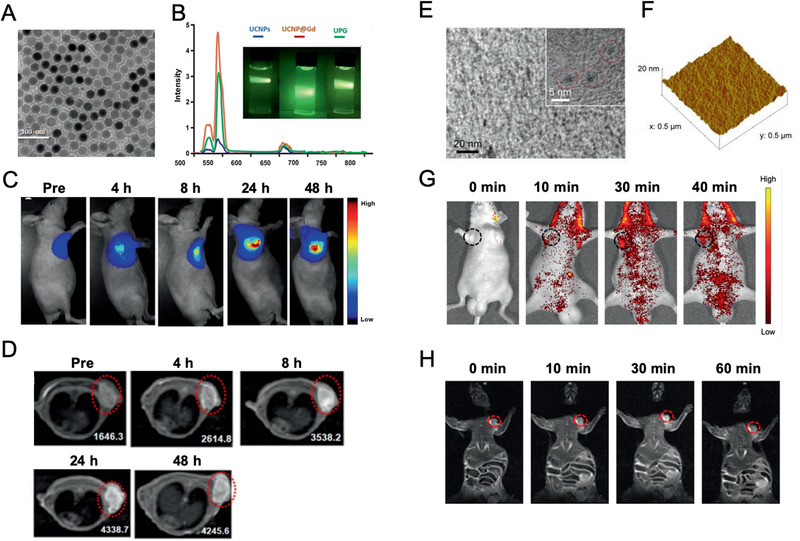
PDAC‐tailored nanomaterials for multimodal imaging. A–D) The TEM image of A) CD326‐grafted UPGs micelles and B) normalized upconversion luminescence spectra (inset: full‐color photographs) under excitation of a 980 nm laser beam. C) The in vivo fluorescence images and D) T1‐weighted MR images of BxPC3 subcutaneous tumor models after i.v. injection of CD326‐grafted UPGs micelles. Reproduced with permission.^[^ ^91^ ^]^ Copyright 2018, Springer Nature. E) The TEM image and F) AFM 3D topographic image of Gd‐Au‐NC‐GPC‐1. G) The in vivo fluorescence images and H) T1‐weighted MR images of COLO‐357 subcutaneous tumor models after i.v. injection of Gd‐Au‐NC‐GPC‐1. Reproduced with permission.^[^ ^92^ ^]^ Copyright 2018, Dove Medical Press, Ltd.

MR contrast agents (e.g., Gd, iron oxide NPs) can be combined with optical imaging agents (e.g., Au NPs, rare‐earth doped NPs) or PET imaging agents, thus providing complementary imaging feature indicative of PDAC. As to Gd‐based nanomaterials, Han et al. synthesized upconversion rare‐earth NPs (UCNP)@Gd^3+^ and encapsulated them into antihuman CD326‐grafted UPGs micelles. The micelles possessed T1‐weighted property (r1, 13.84 × 10^−4^ s^−1^) and exhibited high upconversion luminescence intensity. In fact, both upconversion luminescence (UCL) signal and relative T1 value increased drastically at 8 h after i.v. injection of CD326‐grafted UPGs micelles, implying that the micelles could be served as a powerful tool in PDAC diagnosis (**Figure** **5**A–D).^[^ ^91^ ^]^ Huang et al. synthesized the GPC‐1 antibody conjugated with Gd‐Au NCs (Gd‐Au‐NC‐GPC‐1) for dual‐modal optical imaging/MR imaging. The probe revealed intense red fluorescent emission and strong T1 effect, whose r1 value (17.722 s^−1^ mM^−1^ Gd) was 4 times higher than Gd‐diethylenetriaminepentacetate (DTPA; r1 value = 4.6 s^−1^ mM^−1^ Gd). For Gd‐Au‐NC‐GPC‐1‐treated mice, both fluorescence intensity and T1‐weighted MR signal in tumor sites obviously increased at 30 min post‐injection. Gd‐Au‐NC‐GPC‐1 revealed no obvious biotoxicity to normal cells, proving to be a promising dual‐modal imaging contrast agent for targeted diagnosis of PDAC (Figure 5E–H).^[^ ^92^ ^]^ Moreover, Amirkhanov et al. constructed [^111^In]DOTA_n_‐poly(diamidopropanoyl)^m^‐KRAS2 PNA‐d(Cys‐Ser‐Lys‐Cys) NPs for PET imaging of specific mRNA expression in PDAC cells. They found that the simultaneous administration of Gd‐KRAS2 G12V probe could increase the tumor‐to‐muscle ratios from 3.9 ± 0.4 to 6.3 ± 0.6 in immunocompromised mice bearing human CAPAN2 (G12 V) pancreatic cancer xenografts.^[^ ^93^ ^]^

Iron oxide NPs that can be easily modified with fluorescent dye and tumor‐targeting ligand, have been widely used in MR/optical imaging of PDAC. For example, Wang et al. developed dextran‐coated superparamagnetic iron oxide NPs that were conjugated to Cy5.5 and an uMUC1‐specific peptide. The abundance of uMUC1 is reported to be highly associated with tumor progression and response to chemotherapy. Their changes in uMUC1 levels following GEM chemotherapy were evaluated using optical imaging and T2‐weighted MR imaging before and at 24 h after injection. The results suggested that uMUC1‐targeted imaging could provide a useful approach to predictively assess the therapeutic response of PDAC.^[^ ^94^ ^]^ Besides, Olariu et al. had functionalized iron oxide NPs using Rhodamine B isothiocyanate (RITC) dye and EPCAM antibody to achieve optical and MR imaging of PDAC.^[^ ^95^ ^]^ Moreover, iron oxide NPs could also be conjugated with plectin‐1 antibody and Cy7 (Plectin‐SPION‐Cy7) for MR and fluorescence optical imaging in pancreatic tumor xenografted mice.^[^ ^96^ ^]^

Moreover, US/optical imaging can also be applied to perform anatomic, functional and target specific imaging. Barrefelt et al. introduced air‐filled polyvinyl alcohol microbubbles (PVA‐MBs) and labeled them with a NIR fluorophore VivoTag‐680 for US and optical imaging. Also, the air‐filled PVA‐MBs could overcome the poor vascularization of subcutaneous and orthotopic PDAC xenografts in mice.^[^ ^97^ ^]^

Even though intensive efforts have been made, e.g., various biomarkers have been developed for laboratory test and diverse imaging modalities have been utilized, the early diagnosis of PDAC is still dismal. PDAC‐tailored nanomaterials, especially the nanoassembly strategies provide various possibilities that for example, encompass integration of multiple imaging contrast agents together, selective “turn‐on” performance for specific tumor imaging. Therefore. it can potentially achieve the early diagnosis, monitor the metastasis and improve the prognosis of PDAC.

## 3 PDAC‐Tailored Nanomaterials for Therapeutic Strategies

**Figure 6:**
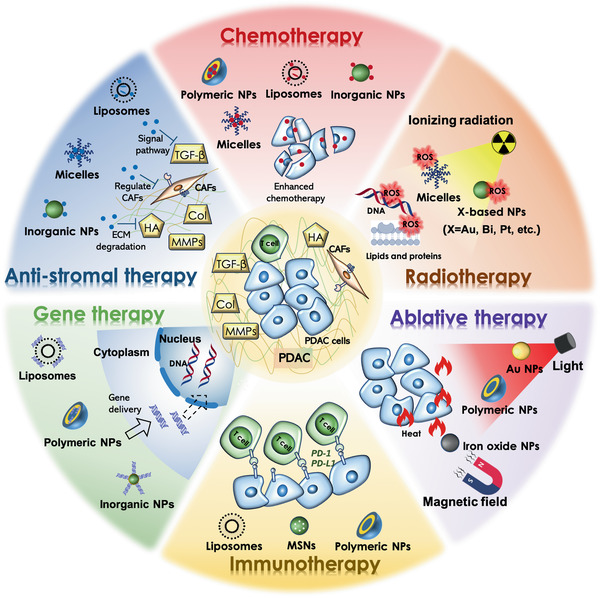
Schematic illustration of various tailor‐made nanomaterials (e.g., liposomes, micelles, polymeric nanomaterials, protein‐based nanomaterials, and inorganic nanomaterials) and their therapeutic modalities (e.g., chemotherapy, anti‐stromal therapy, radiotherapy, gene therapy, immunotherapy, and ablative therapies) for PDAC.

Over the past years, many efforts have been made to enhance the treatment efficiency of PDAC, however, researchers only achieved limited success in improving survival of the patients in clinical studies. One of the merits in the manipulation of the nanomaterials are the convenience in incorporating multiple of unique features, such as enhanced tumor targeting, prolonged circulation, and reduced toxicity. In fact, these benefical nanomaterials are appealing to many researchers and some of them have been successfully used clinically. Recently, a myriad of nanomaterials (e.g., liposomes, micelles, polymeric nanomaterials, protein‐based nanomaterials, and inorganic nanomaterials) that mediated varieties of therapeutic modalities (e.g., chemotherapy, anti‐stromal therapy, radiotherapy, gene therapy, immunotherapy, and ablative therapies) via targeting and/or modulating the unique components/characteristics in PDAC heterogeneous microenvironment will be comprehensively discussed (**Figure** **6**).

### 3.1 PDAC‐Tailored Nanomaterials for Chemotherapy

Chemotherapy remains the mainstay for the treatment of PDAC as most patients with advanced disease are precluded locoregional treatments. However, the efficacy of current chemotherapeutic regimens is limited in improving patient survival in clinical studies. Over the past years, numerous nanomedicine have been attempted for efficient PDAC treatment by enhancing tumor targeting, improving antitumor activity, and reducing the systemic toxicity of chemotherapeutic agents (**Table** **3**). There are number of chemotherapeutic agents proven to be effective on animals models, e.g., 5‐FU,^[^ ^98^ ^]^ GEM,^[^ ^99^ ^]^ irinotecan,^[^ ^100^ ^]^ oxaliplatin,^[^ ^101^ ^]^ marimastat,^[^ ^102^ ^]^ DOX,^[^ ^103^ ^]^ paclitaxel,^[^ ^104^ ^]^ docetaxel,^[^ ^105^ ^]^ withaferin A,^[^ ^106^ ^]^ camptothecin,^[^ ^107^ ^]^ etoposide,^[^ ^108^ ^]^ phospho‐valproic acid,^[^ ^109^ ^]^ salinomycin,^[^ ^110^ ^]^ triptolide, and celastrol.^[^ ^111^ ^]^

**Table 3:** Summary of studies on the nanomaterial‐enabled drug delivery for PDAC therapy

Nanocarriers	Polymers/Macromolecules	Targeting Ligands	Drugs	Cell Lines	In vivo Models	Outcomes	Ref
Micelles	Cetuximab C225‐poly(ethylene glycol)‐block‐poly(2‐methyl‐2‐carboxyl‐propylene carbonategraft‐dodecanol (C225‐PEG‐PCD), GEM conjugated poly(ethylene glycol)‐block‐poly(2‐methyl‐2‐carboxyl‐propylene carbonate‐graft‐dodecanol‐graft‐tetraethylenepentamine) (PEG‐b‐PCC‐g‐GEM‐g‐DC‐g‐TEPA)	Cetuximab C225	GEM, miR‐205	Miapaca‐2 cells	Miapaca‐2 orthotopic tumor model	Enhance EGFR‐mediated cellular uptake, increase accumulation of C225‐micelles, increase apoptosis and reduce EMT	^[^ ^113^ ^]^
Micelles	Poly(ethylene glycol)‐poly(l‐/d‐ glutamate)	–	Cisplatin	BxPC3 cells	BxPC3 subcutaneous tumor model	Promote NPs accumulation and retention, improve antitumor efficacy	^[^ ^114^ ^]^
Micelles	Poly(styrene maleic acid)‐hyaluronic acid (SMA‐HA)	HA	3, 4‐difluorobenzylidene curcumin (CDF)	Miapaca‐2 cells, AsPC‐1 cells	–	Increase the uptake of NPs, reduce CD44 expression, inhibit NF‐KB	^[^ ^306^ ^]^
Polymeric NPs	Dendrigraft poly‐l‐lysine‐EGPLGVRGK‐poly(ethylene glycol)‐poly(caprolactone) (DGL‐EGPLGVRGK‐PEG‐PCL)	–	GEM	Panc‐02 cells, 4T1 cells	Panc‐02/NIH3T3 subcutaneous tumor model, 4T1/NIH3T3 subcutaneous tumor model	Increase long‐term antitumor effect	^[^ ^132^ ^]^
Polymeric NPs	Cell‐penetrating peptide (CPP)‐based amphiphilic peptide (C2KG2R9)‐cholesterol monomers	Human fibroblast activation protein‐*α* (FAP‐*α*) monoclonal antibody (mAb)	DOX	CAFs cells, PC‐3 cells, HUVECs	CAFs/PC‐3 subcutaneous tumor model	Enhance tumor targeting, penetration, and accumulation of various therapeutics, improve the cellular uptake and the antitumor efficacy	^[^ ^146^ ^]^
Polymeric NPs	Chaperonin GroEL	–	DOX	MDA‐MB‐231 cells	MDA‐MB‐231 subcutaneous tumor model, Panc‐1 subcutaneous tumor model	Effective and highly selective drug delivery without adverse effects on the major organs	^[^ ^121^ ^]^
Polymeric NPs	Human serum albumin (HSA)	–	Paclitaxel, tumor necrosis factor (TNF)‐related apoptosis‐inducing ligand (TRAIL)	Miapaca‐2 cells	Miapaca‐2 subcutaneous tumor model	Increase apoptotic activity, improve antitumor efficacy	^[^ ^104^ ^]^
Polymeric NPs	Bovine serum albumin (BSA)		GEM	Miapaca‐2 cells, Panc‐1 cells	–	Enhance cellular uptake and stability of GEM, increase apoptotic activity	^[^ ^99^ ^]^
Polymeric NPs	Aptamer/cell‐penetrating peptide‐camptothecin prodrug	GBI‐10 aptamer	Camptothecin	Miapaca‐2 cells	Miapaca‐2 orthotopic tumor model	Enhance tumor penetration and antitumor efficacy, reduce cytotoxicity	^[^ ^107^ ^]^
Polymeric NPs	Poly(ethylene glycol)‐poly(d,l‐lactic acid) (PEG‐PLA)	–	Salinomycin (SAL)	AsPC‐1 cells	AsPC‐1 subcutaneous tumor model	Increase cell mortality and apoptosis, inhibit invasion and harness EMT, eradicate tumor and increase survival rate	^[^ ^110^ ^]^
Polymeric NPs	Fourth generation poly(amidoamine) (PAMAM) dendrimer‐HA	HA	CDF	Miapaca‐2 cells	–	Enhance cellular uptake, increase in the IC50 value improve safety and therapeutic margin	^[^ ^115^ ^]^
Polymeric NPs	PCL‐CDM‐PAMAM/Pt (G3, G5, G7), PEG‐PCL, PCL	–	Pt prodrug c,c,t‐[Pt(NH_3_)_2_C_l2_(OH)(O_2_CCH_2_CH_2_CO_2_H)]	Panc‐02 cells, MCSs	Panc‐02 orthotopic tumor model	Balance tumor penetration, cell internalization, and tumor retention	^[^ ^116^ ^]^
Polymeric NPs	PEG_2000_‐S‐S‐PLA_6000_, N_3_‐PEG_2000_‐PLA_6000_	MMP‐7	DOX, curcumin	BxPC‐3 cells, AsPC‐1 cells	–	Facilitate the cellular internalization preferentially in the cancer cells, and subsequent nuclear transport	^[^ ^119^ ^]^
NPs‐Liposomes	HSA, dipalmitoylphosphatidylcholine (DPPC), Brij78	–	Ellagic acid (EA)	BxPC‐3 cells	BxPC3/HPaSteC subcutaneous tumor model	Improve drug blood retention, facilitate penetration and accumulation of NPs into tumor matrix, increase apoptosis and inhibit tumor growth	^[^ ^133^ ^]^
NPs‐Gels	Monomethoxy (polyethylene glycol)‐poly(d,l‐lactide‐*co*‐glycolide)‐poly(l‐lysine)‐cyclic peptide (arginine‐glycine‐asparticglutamic‐valine acid) (mPEG‐PLGA‐PLL‐cRGD)	cRGD	Paclitaxel	Aspc‐1 cells, Aspc‐1/PTX cells	Aspc‐1/PTX subcutaneous tumor model, Aspc‐1/PTX orthotopic tumor model	Prolong the release and elimination times, enhance the paclitaxel uptake and the antitumor effects	^[^ ^134^ ^]^
MSNs	DPPC, cholesterol, 1,2‐distearoyl‐sn‐glycero‐3‐phosphoethanolamine‐N‐[methoxy(polyethyleneglycol)] (DSPE‐PEG)	–	Paclitaxel, GEM	Panc‐1 cells	Panc‐1 orthotopic tumor model	Enhance dual delivery carrier efficacy, increase the phosphorylated DNA‐interactive GEM metabolite, decrease the inactivated and deaminated metabolite, inhibit primary tumor growth and eliminated metastatic foci, no local/systemic toxicity	^[^ ^127^ ^]^
MSNs	1,2‐Distearoyl‐sn‐glycero‐3‐phosphocholine (DSPC), cholesterol, DSPE‐PEG	–	Irinotecan	Panc‐1 cells, KPC cells	KPC orthotopic tumor model	Increase drug accumulation at tumor site, treat tumor metastases, improve PDAC survival, decrease toxicity in the gastrointestinal, liver, and bone marrow	^[^ ^100^ ^]^
MSNs	Cancer cell membrane	–	DOX	BxPC3 cells, human pancreatic stellate cells (hPSCs)	BxPC3/hPSCs subcutaneous tumor model	Improve immunoevasion, enhance ECM penetration, tumor accumulation, and antitumor efficacy	^[^ ^128^ ^]^
CdSe/ZnS QDs	MMP‐9 detachable PEG, cathepsin B‐cleavable GEM	cRGD	GEM	BxPC3 cells	BxPC3 subcutaneous tumor model	Increase the accumulation of NP in tumor tissue, enhance the tumor inhibitor activity, reduce the side effects	^[^ ^122^ ^]^
Iron oxide NPs	F127	–	GEM, curcumin	HPAF‐II cells, Panc‐1 cells, pancreatic cancer stem cells (CSCs)	HPAF‐II/PSCs orthotopic tumor model	Increase accumulation and uptake of NPs in tumor site, reduce metastasis and tumor growth	^[^ ^123^ ^]^
Iron oxide NPs	Citric acid	–	Gambogic acid	Capan‐1 cells	–	Induce apoptosis, enhance anticancer activity	^[^ ^129^ ^]^
Metal‐organic frameworks (MOFs)	–	–	GEM	Panc‐1 cells	–	Enhance therapeutic efficiency	^[^ ^124^ ^]^
Calcium phosphosilicate NPs	mPEG	–	5‐FU, GEM	Panc‐1 cells, BxPC‐3 cells	Panc‐1 orthotopic tumor model	Enhance NP/drug delivery and the uptake by tumor cells	^[^ ^125^ ^]^

#### 3.1.1 PDAC‐Tailored Micelles/Polymeric Nanomaterials

Micelles/polymeric nanomaterials prepared from amphiphilic copolymers are widely applied as they have multiple advantages over vector systems in terms of great stability, biocompatibility, easy surface modification, and controlled drug release. The micelles/polymeric nanomaterials can be classified into drug‐encapsulated carriers, polymer‐drug conjugates, and polyion complex micelles.^[^ ^112^ ^]^ For example, poly(ethylene glycol)‐ poly(d,l‐lactic acid) (PEG‐PLA) could form micelles and load drugs such as salinomycin (SAL), a Wnt/*β*‐catenin pathway inhibitor of cancer stem cells (CSCs).^[^ ^110^ ^]^ In addition, Mondal et al. reported on EGFR‐targeting cetuximab (C225) decorated‐cationic complex micelles with GEM and small noncoding RNAs (miR‐205) as the payloads, where miR‐205 can sensitize pancreatic tumor cells to drugs. The complex micelles improved EGFR‐mediated cellular uptake in GEM‐resistant Miapaca‐2 cells and markedly inhibited tumor growth as confirmed in the orthotopic pancreatic tumor model.^[^ ^113^ ^]^ Moreover, Mochida et al. prepared a series of cisplatin‐loaded polymeric micelles (CDDP/m) (l‐, d‐ and d,l‐CDDP/m, respectively), which focused on the difference in the secondary structures of the p(Glu) block. They have emphasized the key role of *α*‐helix bundles of CDDP/m, which exerts longer circulation time to accomplish more appreciable antitumor efficacy than those without secondary structures.^[^ ^114^ ^]^

Besides, well‐designed branching polymers such as poly(amidoamine) (PAMAM) dendrimers having interior cavities and abundant cationic terminal groups, can not only form small‐sized nanocomplexes to load drugs, plasmid DNA, oligonucleotides, and antibodies, but also can be easily modified via PEGylation, acetylation, and glycosylation functionalization.^[^ ^115^ ^]^ For instance, the in vivo performance and antitumor activities of G3‐, G5‐, and G7‐iCluster delivery systems were constructed by using PAMAM dendrimers of different generations (G3‐, G5‐, and G7‐PAMAM) as building blocks. As a result, G5‐PAMAM outperformed the other two counterparts in orthotopic PDAC tumor accumulation and tumor growth inhibition, due to the proper size and particle‐cell interaction.^[^ ^116^ ^]^ However, further study is still needed to decrease the potential cytotoxicity of PAMAM dendrimers due to the abundant cationic groups on their surface.

In recent decades, researchers have focused on biological stimulus‐responsive nanomedicines, which undergo structural and/or chemical changes upon stimuli (pH, redox, hypoxia, enzymes, etc.) to selectively release therapeutic agents.^[^ ^117^ ^]^ For pH‐responsive nanomedicines, Wang and coworkers constructed stimuli‐responsive clustered NPs (iCluster) through the molecular assembly of Pt prodrug‐conjugated PAMAM‐graft‐polycaprolactone with poly(ethylene glycol)‐block‐poly(*ε*‐caprolactone) (PEG‐PCL) copolymer and PCL homopolymer. The iCluster has an initial size of ≈100 nm, which is favorable for long blood circulation and tumor accumulation through tumor vascular fenestrations. Once iCluster accumulated at the PDAC tumor site, the intrinsic tumor extracellular acidity (pH_e_ ≈ 6.5–7.2) would trigger the discharge of Pt prodrug‐conjugated PAMAM dendrimers (≈5 nm) for deep tumor penetration and rapid release of Pt.^[^ ^118^ ^]^ Regarding to redox‐responsive nanomedicines, Mallik group designed redox‐responsive polymersomes using PEG_2000_‐S‐S‐PLA_6000_ that encapsulated with DOX/curcumin, and were conjugated with a MMP‐7 enzyme sensitive peptide (PKKKRKV).^[^ ^119^ ^]^ They further synthesized iRGD peptide‐conjugated hypoxia‐responsive polymer PEG‐azobenzene‐PLA to coload GEM and a STAT3 inhibitor (napabucasin, in clinical trials), which undergo rapid structural destabilization to responsively release two drugs once reaching the hypoxic niches in tumor. Compared with the untreated control group, the polymersomes reduced tumor growth by nearly 250% in mice, and significantly increased necrosis by 60% within the tumors.^[^ ^120^ ^]^ Moreover, He et al. designed a cell‐penetrating peptide‐camptothecin prodrug (Apt/CPP‐CPTD NPs), wherein an ECM component (tenascin‐C) targeting GBI‐10 aptamer was modified onto CPP for in vivo PDAC‐homing. Once the NPs delivered to PDAC stroma, tenascin‐C can effectively detach GBI‐10 aptamer from CPP to free CPP for deep penetration and tumor cell endocytosis. Followed by the internalization of NPs into PDAC cells, the disulfide‐containing CPT prodrug was cleaved under intracellular high redox potential to upregulate the antitumor activity. The in vivo anti‐PDAC results revealed that Apt/CPP‐CPTD NPs had penetrated deeply into the tumor and remarkably inhibited the tumor growth.^[^ ^107^ ^]^ Consequently, stimulus‐responsive nanomedicines may pave the way for precision nanotherapeutics with site‐specific therapeutic manner, improved tissue penetration and cargo delivery efficiency, as well as reduced side effects.

#### 3.1.2 PDAC‐Tailored Protein‐Based Nanomaterials

The natural proteins (e.g., albumin) possessing excellent biocompatibility and biodegradability simultanesouly can be utilized as a relatively effective drug delivery vehicle. Nanoparticle albumin‐bound (nab) technology offers an effective route to deliver hydrophobic chemotherapeutic agents. Regarding broadly applied nab‐paclitaxel, extensive clinical trials of the combinational therapies have been widely studied in patients with locally advanced or metastatic PDAC (**Table** **4**). Besides the commercialized nab‐paclitaxel, nab‐rapamycin (ABI 009) is in phase II trials to treat patients with solid tumors (prostate cancer, NCT00477529; glioblastoma, NCT03463265). Consequently, it is highly required to endow the protein‐based nanomaterials with multifunction (e.g., targeting ability, controlled drug release, or stimuli‐responsiveness) to improve their efficiency and effectiveness in treating PDAC.

**Table 4:** Representative clinical trials of nab‐paclitaxel for patients with PDAC

Condition or disease	Intervention/treatment	Phase	Main Outcomes	Identifier	Ref
Stage IV untreated pancreatic cancer	PEGPH20 + Nab‐paclitaxel + GEM (PAG); Nab‐paclitaxel + GEM (AG)	Phase II	PAG treatment can significantly improve PFS (HR 0.73; 95% CI 0.53‐1.00; *P* = 0.049) for patients with HA‐high tumors (HR 0.51; 95% CI 0.26–1.00; *P* = 0.048).	NCT01839487	^[^ ^141^ ^]^
Locally advanced pancreatic cancer	GEM + Nab‐paclitaxel	Phase II	Among 107 patients, 83 patients achieved disease control (disease control rate 77.6%, 90% CI 70.3–83.5), and 36 patients had a best response to partial response. The overall response rate during induction was 33.6% (90% CI 26.6–41.5).	NCT02301143	^[^ ^307^ ^]^
Stage IV pancreatic cancer	Nab‐paclitaxel + Cisplatin + GEM	Phase II	Among 24 patients, the number of patients considered in CR, PR, SD, PD was 2, 15, 4, and 3, respectively.	NCT01893801	–
Metastatic pancreatic cancer	GEM + Nab‐paclitaxel	Phase I/II	The MTD was 1000 mg m^–2^ of GEM plus 125 mg m^–2^ of nab‐paclitaxel once a week for 3 weeks, every 28 days. Dose‐limiting toxicities were sepsis and neutropenia. At the MTD, the response rate was 48%, with 12.2 median months of overall survival (OS) and 48% 1‐year survival.	NCT00398086	^[^ ^308^ ^]^
Locally advanced or metastatic pancreatic cancer that did not respond to first‐line therapy with GEM	Nab‐paclitaxel	Phase II	Overall survival rate at 6 months was 58% (90% CI 33–76).	NCT00691054	–

Constructed paclitaxel‐bound HSA NPs were embedded with tumor necrosis factor (TNF)‐related apoptosis‐inducing ligand (TRAIL/PTX‐HSA NPs). Compared with plain PTX‐HSA NPs, TRAIL/PTX‐HSA NPs significantly enhanced the antitumor efficiency for more than 25 times in pancreatic Miapaca‐2 cells, resulting from synergistically improving apoptosis and necrosis.^[^ ^104^ ^]^ Besides albumin, chaperonin GroEL can load hydrophobic drugs via an ATP‐switchable double‐layer cage structure and have a specific affinity for protein plectin highly expressed on tumor cell membranes. After GroEL‐DOX got targeted to the tumor, the elevated concentration of ATP within the tumor interstitium can trigger the conformational switch of hydrophobic GroEL, turning into a hydrophilic state for DOX release. As evident from the therapeutic results, there were significant tumor killing efficacy of GroEL‐DOX at both the cellular and animal levels, implying that GroEL can serve as a promising drug delivery vehicle for hydrophobic drug targeting and delivery.^[^ ^121^ ^]^ Although there are many advantages of natural proteins as drug carriers, it is necessary to improve their tumor targeting ability.

#### 3.1.3 PDAC‐Tailored Inorganic Nanomaterials

A wide variety of inorganic nanomaterials are currently under intense development as drug carriers for cancer therapy. Here, MSNs,^[^ ^100^ ^]^ QDs,^[^ ^122^ ^]^ iron oxide NPs,^[^ ^123^ ^]^ metal‐organic framework (MOF) NPs,^[^ ^124^ ^]^ and calcium phosphosilicate NPs,^[^ ^125^ ^]^ have been explored to deliver drugs to pancreatic tumor tissues. For instance, Jia et al. synthesized SiNPs@SiO_2_ with the modification of human fibroblast growth factor‐inducible 14 (FN14)‐glucose (Glu‐FH) and bleomycin (BLM). The above molecularly imprinted polymer NPs (FH‐MIPNPs/BLM) targeted FN14‐overexpressing PDAC cells, and showed a superior therapeutic effect in comparison with the other groups including BLM and saline (tumor volume increased to 1.5× and 2.4×, respectively).^[^ ^126^ ^]^ Nel group developed GEM/PTX‐loaded MSNs with the assistance of supported lipid bilayers (LB), which enhanced GEM loading efficiency to 40 wt%. In addition, the GEM/PTX‐loaded LB‐MSNs increased the phosphorylated DNA‐interactive GEM metabolite up to 13 fold as compared to free GEM. As a result, it inhibited primary tumor growth effectively and eliminated metastatic foci in the Panc‐1 orthotopic model.^[^ ^127^ ^]^ Moreover, they constructed the high‐dose irinotecan loaded LB‐MSNs with lower drug leakage and thus higher safety, compared to the liposomal counterparts.^[^ ^100^ ^]^ Furthermore, Zhang et al. applied BxPC‐3 cells to fabricate cancer cell membrane (CCM)‐coated MSNs with spherical and rod shapes. They found CCM‐coated nanorods (CRs) showed higher endocytosis efficiency than their spherical counterparts, offering improved regulation of the subcellular ER stress and efficient delivery of DOX to the nucleus.^[^ ^128^ ^]^ In addition, Han et al. prepared dual‐enzyme‐sensitive CdSe/ZnS QDs (dQDs) conjugated with MMP‐9 detachable PEG, cathepsin B‐cleavable GEM, and targeting ligand CycloRGD. dQDs achieved a tumor inhibition rate as high as 71.8%, which was much higher than only MMP‐9‐sensitive QDs (mmp‐QDs, 47.8%), only cathepsin B‐sensitive QDs (cb‐QD, 45.4%), and free GEM (21.6%).^[^ ^122^ ^]^ Wang et al. utilized magnetic Fe_3_O_4_ NPs to load gambogic acid by mechanical absorption polymerization. The gambogic acid‐loaded MNP‐Fe_3_O_4_ dramatically enhanced the Bax/Bcl‐2 ratio and the activity of both caspase 9 and caspase 3 in Capan‐1 cells, which efficiently resulted in apoptosis.^[^ ^129^ ^]^

Moreover, several agents such as curcumin,^[^ ^123^ ^]^ deguelin,^[^ ^130^ ^]^ and Hsp90 inhibitors (ICPD47 and ICPD62),^[^ ^131^ ^]^ have been reported to enhance the efficiency of chemotherapy in PDAC cells. For instance, Khan et al. developed a superparamagnetic iron oxide NPs‐based formulation of curcumin (SP‐CUR) to sensitize cells to the standard GEM therapy. The results revealed that SP‐CUR inhibited the crosstalk between tumor cells and stromal cells by suppressing the activation of CXCR4/CXCL12/SHH signaling, thus potentiating GEM therapy.^[^ ^123^ ^]^

#### 3.1.4 PDAC‐Tailored Hybrid Nanomaterials

**Figure 7:**
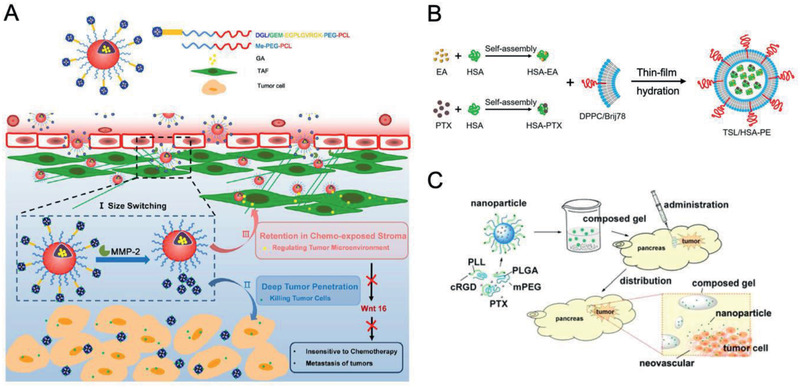
PDAC‐tailored hybrid nanomaterials for chemotherapy. A) Schematic illustration of size‐switchable DGL/GEM@PP/GA hybrid NPs for deep tumor cell killing. Reproduced with permission.^[^ ^132^ ^]^ Copyright 2019, American Chemical Society. B) The synthesis of thermosensitive liposomes (TSL/HSAPE) for enhanced drug penetration and therapeutic efficacy against PDAC. Reproduced with permission.^[^ ^133^ ^]^ Copyright 2017, American Chemical Society. C) Thermosensitive gel composed of PTX‐loaded mPEG‐PLGA‐PLL‐cRGD NPs to reverse drug resistance and realize sustained drug release for PDAC interventional therapy. Reproduced with permission.^[^ ^134^ ^]^ Copyright 2015, American Chemical Society.

The hybrid nanomaterials are rapidly growing and can synergize the advantages of two or more nanoplatforms. For example, after the conjugation of GEM to a small‐sized dendrimer (dendrigraft poly‐l‐lysine, DGL), the synthesized DGL/GEM was then attached to PEG‐PCL via the EGPLGVRGK peptide (a MMP‐2 substrate) for self‐assembly. The assembled nanoparticle complexes can release small‐sized DGL/GEM in response to MMP‐2, which then penetrate into the deep tumor tissue to efficiently kill tumor cell (**Figure** **7**A).^[^ ^132^ ^]^ Moreover, Wei et al. prepared ≈9 nm sized drug‐HSA nanocomplexes, which were then encapsulated into ≈176 nm thermosensitive liposomes (TSL/HSAPE). As the NPs got delivered to the site of tumor, TSL/HSAPE rapidly released small‐sized drug‐HSA nanocomplexes upon the heat treatment, leading to deep matrix penetration and superior tumor growth inhibition (Figure 7B).^[^ ^133^ ^]^ In addition, nanogels can serve as a reservoir for NPs to accomplish sustained release profiles and long‐term retention in tumors. Shen et al. synthesized monomethoxy (polyethylene glycol)‐poly(d,l‐lactide‐*co*‐glycolide)‐poly(l‐lysine)‐cyclic peptide (arginine‐glycine‐asparticglutamic‐valine acid) (mPEG‐PLGA‐PLL‐cRGD) to obtain paclitaxel‐loaded mPEG‐PLGA‐PLL‐cRGD NPs, which were then encapsulated with thermosensitive gels. NPs‐Gels retained in the site of tumor for over 50 d and exhibited a much higher inhibition rate (at the low dose of paclitaxel ≈250 µg kg^−1^) in vivo than NPs or Gels alone (Figure 7C).^[^ ^134^ ^]^

Above all, tailor‐made fabrication and delivery efficiency for PDAC improved the tumor targeting and therapeutic efficacy of chemotherapeutic agents. We believe that much more advanced nanomaterials could to be developed for PDAC treatment with a focus on drug resistance and the long term toxicity of nanomaterials in the future.

### 3.2 PDAC‐Tailored Nanomaterials for Anti‐stromal Therapy

Despite the great hope for nanotechnology‐enabled chemotherapies, the desmoplastic stroma and hypovascular nature of PDAC leads to the insufficient accumulation of nanomaterials in tumor tissues, resulting in limited clinical benefit.^[^ ^135^ ^]^ The stroma contains various ECM components such as well‐organized fibrillar macromolecules, including collagens (type I and type IV), hyaluronic acid (HA), fibronectin, tenascin C, and versican. The complex and highly dynamic network of the ECM is constantly degraded by enzymes (e.g., MMPs and collagenases, etc.) and supplemented by CAFs secretion.^[^ ^136^ ^]^ In relation to CAFs activation and ECM production, prostromal factors including the transforming growth factor‐*β* (TGF‐*β*), Hedgehog (HH), basic fibroblast growth factor (bFGF), platelet‐derived growth factor (PDGF), and interleukin (IL) have been recognized to play major roles.^[^ ^137^ ^]^ Hence, the deposition of ECM to the developing pancreatic tumor may lead to a high association between CAFs and PDAC cells. Thus, they abnormally create a tumor promoting microenvironment to facilitate local tumor growth, distant metastasis and drug resistance.^[^ ^138^ ^]^ Therefore, we have summarized the recent advances in PDAC‐tailored nanomaterials for PDAC via targeting ECM, CAFs and prostromal signaling are summarized in **Table** **5**.

**Table 5:** Summary of studies on the PDAC‐tailored nanomaterials for anti‐stromal therapy

Nanocarriers	Polymers/Macromolecules	Drugs	Function	Cell Lines	In vivo Models	Outcome	Ref
Liposomes	1,2‐dimyristoyl‐sn‐glycero‐3‐phosphocholine (DMPC), DSPE‐PEG_2000_, cholesterol	Collagenase type‐I	Disassemble the dense collagen stroma	KPC cells	KPC orthotopic tumor model	Degrade the ECM, increase drug penetration into tumor and improve PDAC treatment	^[^ ^139^ ^]^
Liposomes	Amphiphilic peptide with the MMP‐2‐specific cleavable sequence (GPLGIAGQ), DSPE‐PEG_2000_, l‐*α*phosphatidylcholine	Pirfenidone	Downregulate the multiple components of ECM expressed by the PSCs	Miapaca‐2 cells, PSCs	Miapaca‐2/PSCs subcutaneous tumor model	Increase the penetration of GEM into the tumor tissue, enhance the efficacy of GEM for tumor treatment	^[^ ^152^ ^]^
Liposomes	MMP‐2‐specific cleavable DSPE‐PEG_3400_‐pep(CSSSGPLG‐IAGQSSS)‐*β*‐cyclodextrin (*β*‐CD), DSPE‐PEG_3400_‐RGD, cholesterol, Brij 35	Pirfenidone	Downregulate the multiple components of ECM expressed by the PSCs	Panc‐1 cells	Panc‐1/PSCs subcutaneous tumor model	Increase drug perfusion, enhance therapeutic efficacy, no overt side effects	^[^ ^153^ ^]^
NPs	PEG‐PLA	Fraxinellone	Regulate TGF‐*β* signaling	Panc‐1 cells, NIH3T3 cells	Panc‐1/NIH3T3 subcutaneous tumor model, Panc‐1/NIH3T3 orthotopic tumor model	Enhance tumor blood perfusion and internalization by the tumor cells, significantly prolong survival	^[^ ^154^ ^]^
NPs	Dendrigraft poly‐l‐lysine‐EGPLGVRGK‐poly(ethylene glycol)‐poly(caprolactone) (DGL‐EGPLGVRGK‐PEG‐PCL)	18*β*‐Glycyrrhetinic acid (GA)	Regulate the activated TAFs and collagen	Panc‐02 cells	Panc‐02/NIH3T3 subcutaneous tumor model	Increase NPs accumulation around the tumor vessel, significant long‐term antitumor effect	^[^ ^132^ ^]^
NPs	PEG_5000_‐PCL_5000_	GDC‐0449	Inhibit the fibroblast‐induced upregulation of SHH signaling‐related proteins	BxPC‐3 cells, SW1990 cells, NIH3T3 cells	BxPC‐3 subcutaneous tumor model	Downregulate SHH signaling proteins, enhance antitumor efficacy	^[^ ^170^ ^]^
NPs‐Liposomes	HSA, DPPC, Brij78	Ellagic acid (EA)	Inhibit PSCs proliferation	BxPC‐3 cells	BxPC3/HPaSteC subcutaneous tumor model	Improve drug blood retention, facilitate tumor matrix penetration and accumulation of NPs, increase apoptosis, inhibit tumor growth	^[^ ^133^ ^]^
Micelles	Anionic polymer consisted of a brushlike PEG block and a brushlike PCL block	Cyclopamine (CPA), paclitaxel, anti‐PD1	Disrupt stroma, increase the intratumoral vasculature density, and promote the tumor infiltration by cytotoxic CD8^+^ T cells	KRAS cells	KRAS orthotopic tumor model	Enhance tumor infiltration of CD8^+^ T cells, increase antitumor efficacy, prolong animal survival	^[^ ^171^ ^]^
Micelles	Block copolymer consisted of poly[oligo(ethylene glycol) monomethyl ether methacrylate]_31_‐block‐poly(2‐hydroxyethyl methacrylate)_26_ (p(OEGMA)_31_‐b‐p(HEMA‐CL_5_)_26_) and triethoxysilane functional groups	CPA	Disrupt stroma and enhance radiation response	Miapaca‐2 cells, L3.6pl cells, Panc‐1 cells, hPSCs	–	Increase cytotoxicity, enhance the radiation therapy of Cs‐137, promote tumor and tumor‐associated stroma treatment	^[^ ^209^ ^]^
Micelles	AE105 peptide modified polyethylene glycol‐polyarginine‐polylysine (PEG‐pArg‐pLys)	PTX	Alter collagen architecture and eliminate CAF	Miapaca‐2 cells, Panc‐02 cells	Miapaca‐2 orthotopic tumor model	Promote effective drug delivery, enhance the antitumor effectiveness of chemotherapeutics, maintain the neighbor suppression effect, prevent tumor metastasis	^[^ ^148^ ^]^
Iron oxide NPs	–	Relaxin‐2 (RLX)	Inhibit the TGF‐*β*‐induced PSCs differentiation into CAF‐like myofibroblasts	Panc‐1 cells, hPSCs	Panc‐1/hPSCs subcutaneous tumor model	Inhibit tumor growth, enhance tumor therapy	^[^ ^143^ ^]^
Iron oxide NPs	PEG‐COOH	FGF2	Inhibit the TGF‐*β*‐induced PSCs differentiation into CAF‐like myofibroblasts	Panc‐1 cells, hPSCs	Panc‐1/hPSCs subcutaneous tumor model	Enhance the effect of GEM	^[^ ^144^ ^]^
MSNs	Polyethyleneimine‐polyethylene glycol (PEI‐PEG)	LY364947	Inhibit TGF‐*β* signaling pathway	BxPC3 cells	BxPC‐3 subcutaneous tumor model	Facilitate biodistribution and retention at the tumor site, enhance drug delivery at the PDAC tumor site and shrinkage of the tumor xenografts	^[^ ^166^ ^]^
Iron oxide NPs	Peptide	Metformin	Downregulate the multiple components of ECM expressed by the PSCs	Panc‐1 cells	Panc‐1/hPSCs subcutaneous tumor model, Panc‐1/hPSCs orthotopic tumor model	Inhibit the generation of *α*‐SMA and collagen, growth inhibition ratio ≈91.2% over 30 d of treatment	^[^ ^157^ ^]^
Au NPs	–	–	Disrupt PDAC cells‐PSCs crosstalk	AsPc1 cells, Panc‐1 cells, CAF19 cells, iTAF cells	AsPc1 orthotopic tumor model, AsPc1/CAF19 orthotopic tumor model	Inhibit matrix deposition, enhance angiogenesis, reprogram the TME, inhibit tumor growth	^[^ ^160^ ^]^

#### 3.2.1 Facilitating ECM Degradation

Some studies focused on the direct disintegration of the dense PDAC stroma (e.g., collagen and HA) for improving drug penetration into the pancreatic tumor. For instance, collagenase type‐I with specificity toward collagen fibers was encapsulated in a liposome (≈100 nm, termed as a collagozome) that provided protection for the collagenase from premature deactivation and ensured the drug delivery to tumor sites. Masson's Trichrome staining result demonstrated reduced collagen levels in tumors tissues by 43% in collagozome group compared to the empty liposome group, and by 31% in the free collagenase group. Moreover, the combination of collagozome and paclitaxel micelles decreased pancreatic tumor size by 87% in comparison to empty liposomes plus paclitaxel micelles.^[^ ^139^ ^]^

Considering the excessive HA accumulation in PDAC,^[^ ^140^ ^]^ PEGPH20, a pegylated recombinant human hyaluronidase, could degrade HA to reduce interstitial gel‐fluid pressure, improve vascular perfusion, and increase access to anticancer therapies. Moreover, the randomized phase II study of PEGPH20 plus nab‐paclitaxel/GEM (PAG) versus nab‐paclitaxel/GEM in patients demonstrated that PAG treatment significantly improved overall progression‐free survival (PFS) (hazard ratio (HR), 0.73; 95% CI, 0.53–1.00; *P* = 0.049) for patients with HA‐high tumors (HR, 0.51; 95% CI, 0.26–1.00; *P* = 0.048). The objective response rate of patients with HA‐high tumors was 45% versus 31% (PAG v AG), and median overall survival was 11.5 versus 8.5 months (HR, 0.96; 95% CI, 0.57–1.61), indicating the great therapeutic potential of PAG treatment in patients with HA‐high metastatic PDAC (NCT01839487).^[^ ^141^ ^]^

In addition to the induction of exogenous ECM‐degrading enzymes, endogenous hormones can also be utilized to degrade ECM. For example, relaxin‐2 (RLX), an endogenous hormone, can decrease collagen production while promoting collagen degradation via the inhibition of TGF‐*β*/Smad3 signaling.^[^ ^142^ ^]^ As reported by Mardhian et al., RLX‐iron oxide NPs significantly inhibited TGF‐*β*‐induced PSCs differentiation via suppressing pSmad2 signaling pathway to downregulate collagen I, desmin and CD31 expression, and ultimately retarded the tumor growth and potentiated the chemotherapy effect of GEM.^[^ ^143^ ^]^ They further paid attention to FGF2 inhibitory effects against TGF‐*β* via Smad2/3 and ERK1/2 signaling pathways, and covalently conjugated FGF2 to PEGylated dextran‐coated iron oxide NPs (FGF2‐IONPs). Hence, functionalized FGF2‐IONPs have significantly downregulated the *α*‐SMA and collagen‐1 expression to reduce the stroma barrier, resulting in enhanced GEM efficacy.^[^ ^144^ ^]^

#### 3.2.2 Targeting CAFs

Active CAFs proliferate and generate huge amounts of ECM components, which further facilitate tumor growth, chemoresistance, and immune tolerance. Moreover, CAFs are localized at perivascular space and thus could be served as a promising therapeutic target of PDAC. Interestingly, modulating the activity of CAFs, such as eliminating them, reducing their activity, or directly inducing them quiescent, is an attractive strategy and holds the potential in anti‐stromal therapy.

##### Eliminating CAFs

The specific markers of CAFs offer a potent strategy to eliminate CAFs with high specificity. Fibroblast activation protein (FAP), a membrane‐bound serine protease, is selectively overexpressed by CAFs and closely associated with tumor progression and poor prognosis.^[^ ^145^ ^]^ Nie group delivered chemotherapeutic drugs into CAFs and other stromal cells for directly depleting CAFs and overcoming the stromal barriers. They have developed CPP‐based amphiphilic peptide (C2KG2R9)‐cholesterol monomers that can self‐assemble into peptide NPs (PNP) and encapsulate DOX (PNP‐D). The addition of mouse monoclonal antibody (mAb) molecules allows PNP‐D‐mAb to specifically target the highly expressed human FAP‐*α* on CAFs, and subsequently exhibit the superior tumor‐penetrating ability and therapeutic effect.^[^ ^146^ ^]^

As reported, nab‐paclitaxel can alter collagen architecture and eliminate CAFs by the generating reactive oxygen species (ROS) rather than the chemotherapeutic effect.^[^ ^147^ ^]^ Afterward, it augments cytotoxic T cell response and decreases regulatory T cells (Tregs) as the main tumor immunosuppressive cells. Chen et al. loaded paclitaxel and monophosphorylated GEM into AE105 peptide modified pH‐sensitive micelles (called T‐RKP micelles). After targeting uPAR expressed in the pancreatic tumor, T‐RKP micelles disrupted the internal tumor stroma and thereby promoted the antitumor effectiveness, preserving the external stroma to prevent tumor metastasis. The death of tumor cells further led to the activation of T cells to kill more tumor cells.^[^ ^148^ ^]^ Furthermore, Lv et al. combined paclitaxel with MMPs inhibitor marimastat (MATT) to suppress tumor growth and inhibit metastatic spread. They have synthesized self‐assembled HA‐paclitaxel (HA‐PTX) prodrugs and MATT‐loaded thermosensitive liposomes (MATT‐LTSLs) into hybrid NPs (called HA‐PTX/MATT‐LTSL HNPs). Evidently, HA‐PTX/MATT‐LTSL HNPs inhibited the expression of MMP (>fivefold) and blocked the fibroblast activation by downregulating the TGF‐*β*1 expression up to fivefold. Thus, NPs have significantly improved deep tumor penetration and inhibited tumor growth.^[^ ^102^ ^]^

However, some studies have reported that CAFs depletion is likely to be associated with impaired immune response, rapid tumor progression and reduced survival. For example, some components of PDAC stroma such as HH inhibit tumor growth in part by restraining tumor angiogenesis.^[^ ^149^ ^]^ In addition, increased CD4^+^ Foxp3^+^ Tregs and suppressed immune surveillance were observed in myofibroblast‐depleted mouse tumors.^[^ ^150^ ^]^ Herein, the ongoing efforts are essential to elucidate the change of PDAC microenvironment during the killing of CAFs. Moreover, combination therapies involving immunotherapy and gene therapy might offer insights for achieving effective and safe anti‐stromal therapy of PDAC.

##### Reducing CAFs Activity

**Figure 8:**
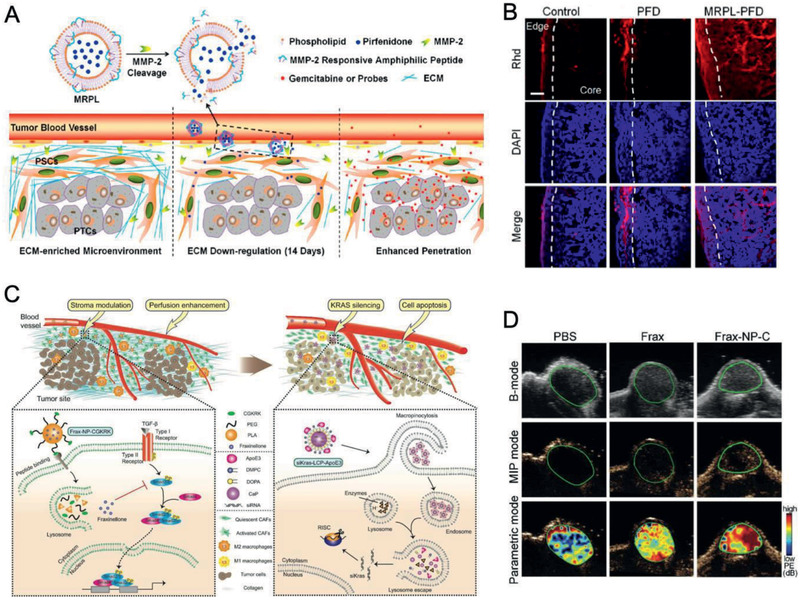
PDAC‐tailored nanomaterials for anti‐stromal therapy. A) Schematic illustration of PFD loaded MMP‐2 responsive peptide hybrid liposome (MRPL‐PFD) to downregulate ECM and enhance drug penetration for improved PDAC therapy. B) Rhd penetration in PSCs/Miapaca‐2 subcutaneous tumor models after the treatment for 2 weeks. Blue, DAPI. Red: Rhd. Dotted lines: border between edge and core of tumor tissue. Scale bar, 100 µm. Reproduced with permission.^[^ ^152^ ^]^ Copyright 2017, American Chemical Society. C) Schematic illustration of fraxinellone‐loaded CGKRK‐modified NPs (Frax‐NP‐CGKRK) to attenuate the dense stroma and improve tumor blood perfusion. D) B‐mode, maximum intensity persistence (MIP) mode, and pseudocolor parametric mode images of Panc‐1/NIH3T3 subcutaneous tumor models treated with PBS, Frax, and Frax‐NP‐CGKRK. Reproduced with permission.^[^ ^154^ ^]^ Copyright 2019, Wiley‐VCH.

Besides eliminating CAFs, the inactivation of CAFs is also proposed to reduce ECM production. Some antifibrotic agents, such as pirfenidone, fraxinellone, ellagic acid (EA), 18*β*‐glycyrrhetinic acid (GA), and metformin are applied to regulate the activities of CAFs, hence promoting the antitumor efficacy of drugs. For example, pirfenidone as an antifibrotic agent can inhibit PSCs and tumor‐stromal interaction in PDAC.^[^ ^151^ ^]^ With the benefit of nanotechnology, Nie group loaded pirfenidone into an MMP‐2 responsive peptide‐hybrid liposome (termed as MRPL‐PFD). When it got delivered to the pancreatic tumor environment with abundant MMPs, MRPL‐PFD was responsively triggered to release pirfenidone followed by downregulation of multiple ECM components expressed by the PSCs. Therefore, MRPL‐PFD improved the penetration of GEM into the tumor tissue and enhanced the therapy efficacy for pancreatic tumors (**Figure** **8**A,B).^[^ ^152^ ^]^ Additionally, they developed a *β*‐cyclodextrin (*β*‐CD) and RGD modified MMP‐2 responsive liposome (called LRC‐GEM‐PFD), where pirfenidone and GEM were encapsulated into *β*‐CD and inside the liposomes, respectively. LRC‐GEM‐PFD would be disintegrated into PFD‐loaded *β*‐CDs and GEM liposome via the cleavage of an MMP‐2 responsive peptide. By downregulating collagen I and TGF‐*β* for more than twofold as compared to free pirfenidone, LRC‐GEM‐PFD improved drug penetration and chemotherapeutic efficacy.^[^ ^153^ ^]^

Pei et al. developed antifibrotic fraxinellone‐loaded CGKRK‐modified NPs (Frax‐NP‐CGKRK) that could recognize heparan sulfate proteoglycan overexpressed in the TME. Through successfully inhibiting pSmad2/3 signals in a TGF‐*β*/Smad pathway, Frax‐NP‐CGKRK reversed the activated CAFs and decreased M2 macrophages to attenuate the collagen deposit (Figure 8C,D).^[^ ^154^ ^]^

GA imparts a significant effect on the down‐regulation of Wnt 16 that is a major damage response program (DRP) molecule and promotes rapid tumor cell metastasis and resistance to chemotherapy.^[^ ^155^ ^]^ Cun et al. utilized GA molecules and developed an MMP‐2 sensitive size‐switchable NPs (DGL/GEM@PP/GA). As the NPs accumulated at the MMP‐2 overexpressed tumor, GA‐encapsulated large NPs (PP/GA) were triggered to be separated with GEM‐conjugated small dendrimers (DGL/GEM). The PP/GA accumulated around tumor vessels and significantly suppressed the expression of Wnt 16 and *α*‐SMA in GEM‐treated activated NIH3T3 cells. Apparently, DGL/GEM@PP/GA displayed significant and long‐term antitumor effect in stroma‐rich PDAC via CAFs regulation and deep tumor penetration.^[^ ^132^ ^]^

Furthermore, EA has been reported to efficiently inhibit PSCs proliferation.^[^ ^156^ ^]^ Wei et al. coencapsulated EA‐HSA complexes and paclitaxel‐HSA complexes into thermosensitive liposomes (TSL/HSAPE). As it got delivered to locally heated tumors, TSL/HSAPE rapidly released drug‐HSA complexes, which modulated PSCs by disrupting PDAC cells‐PSCs interaction, and finally induced tumor growth inhibition and apoptosis in vivo.^[^ ^133^ ^]^

Recently, Jin and Gao group applied metformin (MET) to suppress the activity of PSCs via downregulating the expression of TGF‐*β*, and thus inhibit the generation of *α*‐SMA and collagen in the dense stroma of PDAC. It promoted the delivery of NPs and remarkably improved therapeutic efficacy with a growth inhibition ratio up to 91.2% over 30 d of treatment.^[^ ^157^ ^]^

These studies highlight the potential of CAFs inactivation by antifibrotic agents‐based nanomaterials. In general, this strategy is mild and transient, and more importantly, much safer than the CAFs elimination strategy.

##### Inducing CAFs Quiescent

Since quiescent fibroblasts can be activated into CAFs for ECM generation and tumor progression, reversing activated CAFs back to a resting phenotype might be a direct way for anti‐stromal therapy. ATRA downregulates the release of active TGF‐*β* from PSCs, thus keep PSCs out of an active state and a tumor‐favoring stiff microenvironment.^[^ ^158^ ^]^ ATRA in combination with GEM can mediate a range of signaling cascades (Wnt, hedgehog, retinoid, and FGF) in the tumor‐stroma cross‐talk.^[^ ^159^ ^]^ More interestingly, inorganic Au NPs were reported to alter the secretory profile of PDAC cells and PSCs, disrupt the perpetual activation of PSCs and the PDAC cells‐PSCs crosstalk. Hence, Au NPs have effectively inhibited the matrix deposition and thus suppressed tumor growth in the orthotopic coimplantation model.^[^ ^160^ ^]^

#### 3.2.3 Targeting Prostromal Signaling

Quiescent fibroblasts would be activated into CAFs via the endothelial‐mesenchymal transition (EMT), which are induced by paracrine cytokines secreted from tumor cells, including TGF‐*β*, PDGF, FGF, SHH, and IL‐6.^[^ ^137^ ^]^ Once CAFs are activated, they continuously secrete various cytokines (e.g., TGF‐*β*, PDGF, FGF, CXCL12, IL‐6, and connective tissue growth factor (CTGF), etc.), which further maintain the activated phenotype via autocrine pathways and stimulate tumor cells via paracrine pathways.^[^ ^136^ ^]^ Therefore, interfering with these signaling pathways by scavenging prostromal cytokines, blocking receptor activation and inhibiting downstream signaling, serves as a feasible strategy for anti‐stromal therapy.

##### Scavenging Prostromal Cytokines

The introduction of small trapping proteins could allow deeper diffusion into tumors, enhancing topical therapeutic outcomes. IL‐10 is an immunosuppressive cytokine, whose upregulation is strongly correlated with tumor progression.^[^ ^161^ ^]^ Huang group constructed lipid‐protamine‐DNA (LPD) NPs loaded with trap genes (IL‐10 trap and CXCL12 trap), which reduced immunosuppressive cells and activated immunosuppressive tolerogenic dendritic cells. This LPD NPs effectively inhibited tumor growth by 20% after treatments in a preclinical allograft PDAC model.^[^ ^162^ ^]^ This strategy might be useful to change the immunosuppressive microenvironment in the tumor to prevent PDAC invasion and prolong patient survival.

##### Blocking Receptor Activation

Developing nanomaterials to pharmacologically block receptor activation reveals numerous advantages in anti‐stromal therapy. CXCL12 is mainly excreted by CAFs and interacts with its cognate receptor C‐X‐C‐motif chemokine receptor 4 (CXCR4) for initiating and maintaining the transdifferentiation of fibroblasts into activated CAFs.^[^ ^163^ ^]^ The disruption of the CXCL12/CXCR4 axis can block the evolution of CAFs and increase immune cell infiltration in desmoplastic tumors.^[^ ^164^ ^]^ Huang group constructed liposome‐protamine‐DNA with plasmids encoding small trapping proteins targeting CXCL12 and PD‐L1 (termed pCombo trap NPs). pCombo trap NPs significantly shrunk the tumor volume, prolonged survival, and reduced metastasis of PDAC. They also found that the CXCL12 trap allowed T cell penetration into tumor sites, and PD‐L1 trap accelerated T cell infiltration to kill PDAC cells.^[^ ^165^ ^]^ In addition, Nel group focused on LY364947 (a small molecule TGF‐*β* inhibitor) that owns nitrogen atoms and thus can efficiently attach to polyethyleneimine (PEI) amines through H‐bonding. They then loaded LY364947/GEM on PEI‐PEG‐MSNs to overcome stromal resistance, and thereby achieve GEM delivery and effective shrinkage of the PDAC tumor xenografts.^[^ ^166^ ^]^ It would be worthy to study whether the nanomaterials could inhibit the activation and secretion of CAFs.

##### Inhibiting Downstream Signaling

As to the SHH signaling pathway, there are overexpressed factors including SMO, PTCH1 and Gli‐1 associated with poor prognosis of PDAC.^[^ ^167^ ^]^ The blockage of SHH can not only inhibit the proliferation of tumor cells but also disrupt stroma and promote chemotherapeutic drug delivery.^[^ ^168^ ^]^ IPI‐926 is a specific inhibitor of the prostomial SHH signaling cascade and could deplete the dense desmoplastic mesenchymal network to significantly reduce tumor stroma in PDAC. Unfortunately, IPI‐926 plus GEM remained unsatisfactory to offer a significant survival benefit in phase I and phase II trials, possibly due to the promoted vascularization and invasion of the tumor.^[^ ^169^ ^]^ In addition, Zhou et al. combined commercial SHH inhibitor GDC‐0449 with nanosized PEG‐PCL‐DOX micelles. The GDC‐0449/PEG‐PCL‐DOX synergistically reversed fibroblast‐induced DOX resistance in SMO‐positive PDAC cells and inhibited BxPC‐3 tumor growth, via downregulating SHH signaling proteins and reducing tumor stroma density.^[^ ^170^ ^]^ Besides, cyclopamine (CPA), another SHH inhibitor was codelivered with paclitaxel into a polymeric micelle‐based nanoformulation (M‐CPA/PTX). M‐CPA/PTX remodeled the PDAC stroma by increasing the intratumoral vasculature density and thus promoted the tumor infiltration by cytotoxic CD8^+^ T lymphocytes (CTLs). The combination of M‐CPA/PTX and the PD‐1 checkpoint blockade significantly contributed to prolonged animal survival and superior antitumor efficacy.^[^ ^171^ ^]^

Despite many inspiring effects in anti‐stromal therapy, more ECM molecules that are involved in mechanical remodeling of the microenvironment and modulating tissue responses, remain to be considered as potential targets for effective PDAC therapy. For example, caveolin‐1 (Cav‐1) is the main constituent of the caveolae membrane domain, whose high expression in CAF favors tumor progression via regulating TME and thus promotes the resistance to therapies.^[^ ^172^ ^]^ Consequently, targeting Cav‐1 and other ECM biomarkers might serve as an important therapeutic option to prevent the invasion and metastasis of PDAC. Moreover, potential adverse effects during anti‐stromal therapy especially the risk of increasing tumor metastasis cannot be ignored. To ultimately eradicate PDAC, we can focus on developing multifunctional nanomaterials for synergistic combinations of anti‐stromal therapy and other effective treatments such as immunotherapy.

### 3.3 PDAC‐Tailored Nanomaterials for Gene Therapy

Even with the presence of signaling pathway targeting‐based treatments (e.g., SHH inhibitors), and DNA repair pathways‐based treatments (e.g., poly(ADP‐ribose) polymerase (PARP) inhibitors), the results either indicates poor prognosis or fails in survival improvement for PDAC patients.^[^ ^149^, ^173^ ^]^ In 2018, Patisiran (ONPATTRO, Alnylam) was approved by US Food and Drug Administration (FDA) for the treatment of hereditary ATTR (hATTR) amyloidosis, which is the first‐of‐its‐kind small interfering RNA (siRNA) drug in the whole 20 years since the discovery of the RNA interference (RNAi) phenomenon. This event brings the concept of gene therapy from laboratory research to clinical reality and encouragingly opens the year of Nucleic Acids Nanomedicine. In addition, it raises the hope for complete cure of PDAC, rather than only slowing its progression.

In fact, remarkable advances have been made in gene therapy for PDAC. RNAi has emerged as a promising method that regulates target genes to achieve sequence‐specific gene silencing, inducing the degradation of complementary messenger RNA (mRNA) and inhibition of target protein production.^[^ ^174^ ^]^ There are different types of RNAi molecules such as microRNA (miRNA), small‐interfering (siRNA) and short hairpin RNA (shRNA). Nevertheless, their easy degradation during blood circulation and the poor precision in delivery to the desired target cells require the development of safe and effective nanocarriers for RNAi‐based therapies.^[^ ^175^, ^176^ ^]^ Cationic polymers including polyethylenimine (PEI), poly‐l‐lysine (PLL) and cyclodextrin‐based polycation, as well as lipid‐based nanocarriers, have been widely used to deliver RNAi molecules. Moreover, inorganic nanomaterials, such as Au NPs, MSNs, CNTs, QDs, and so on, are novel alternatives that also offer tremendous opportunities for RNAi therapies (**Table** **6**).^[^ ^176^ ^]^

**Table 6:** Summary of studies on the PDAC‐tailored nanomaterials for gene therapy

Gene	Gene Function	Drug Codelivery	Nanocarriers	Polymers/Macromolecules	Cell Lines	In vivo Model	Outcome	Ref
Anti‐miR‐21	Regulate PDAC initiation and progression	–	NPs	Fatty acid groups‐iRGD	Panc‐1 cells, BxPC3 cells, PL‐45 cells	Patient‐derived‐organoid (PDO) avatars and PDX avatars	Decrease tumor cell growth	^[^ ^191^ ^]^
TUBB3/*β*III‐tubulin siRNA	Regulate tumor growth and metastases	–	Polymeric NPs	Star polymers with cationic poly(dimethylaminoethyl methacrylate) (PDMAEMA) side‐arms and POEGMA	Miapaca‐2 cells, HPAF‐II cells	Miapaca‐2 subcutaneous tumor model, Miapaca‐2/HPAF‐II orthotopic tumor model	Enhance delivery and accumulation of siRNA to tumors, no toxicity to normal cells	^[^ ^193^ ^]^
Anti‐miR‐210; siKRAS^G12^D	Inactivate PSCs, kill PDAC cells	AMD3100	Polymeric NPs	Cholesterol‐conjugated polymeric AMD3100	KPC8060 cells, PSCs	KPC8060 orthotopic tumor model	Deplete stroma, reduce immunosuppression, delay tumor growth, inhibit metastasis, prolong survival	^[^ ^178^ ^]^
siKRAS	Kill PDAC cells	–	Calcium phosphate NPs	High‐density lipoprotein ApoE3	Panc‐1 cells, NIH3T3 cells	Panc‐1/NIH3T3 subcutaneous tumor model, Panc‐1/NIH3T3 orthotopic tumor model	Enhance tumor blood perfusion and uptake of NPs by the tumor cells, prolong survival	^[^ ^154^ ^]^
siKRAS	Kill PDAC cells	DOX,	Graphene quantum dots (GQDs)	Cationic polylactides with pendant tertiary amine groups	Miapaca‐2 cells	–	Keep significantly stable in physiologically mimicking media, promote downregulation of KRAS, enhance anticancer activity	^[^ ^179^ ^]^
HIF1a siRNA	Regulate tumor invasion, proliferation, angiogenesis, and drug resistance	GEM	Polymeric NPs	Lipid‐polymer hybrid nanoparticles using mPEG‐ polyethylenimine‐PLGA, *ε*‐pLys, lecithin	Panc‐1 cells	Panc‐1 subcutaneous tumor model, Panc‐1 orthotopic tumor model	Improve drug release, suppress the HIF1*α* expression, enhance tumor vasculature efficacy, inhibit tumor metastasis	^[^ ^181^ ^]^
EPAS1 siRNA	Regulate tumor cell adaption to a hypoxic microenvironment, and thus suppress tumor growth	–	Polymeric NPs	PLGA‐poloxamer NPs	BxPC‐3 cells	BxPC‐3 subcutaneous tumor model	Induce cell apoptosis, inhibit the microvessel formation and tumor growth	^[^ ^182^ ^]^
NGFs siRNA	Regulate density of neurites, and thus suppress tumor growth	–	Au NCs	–	Panc‐1 cells	Panc‐1 subcutaneous tumor model, Panc‐1 orthotopic tumor model, PDX tumor model	Increase the siRNA stability, cellular uptake and tumor accumulation of siRNA, prolong the circulation lifetime of siRNA, inhibit tumor progression	^[^ ^183^ ^]^
Anti‐ITCH siRNA, shRNA	Sensitize chemotherapy	GEM	Dendrimers	Generation 3 polypropylenimine dendrimer (DAB‐Am16)	Miapaca‐2 cells, Panc‐1 cells, HPAC cells, BxPC3 cells	Miapaca‐2 subcutaneous tumor model	Downregulate DAB‐Am16/shRNA mediated ITCH, enhance the sensitization of cancer to GEM	^[^ ^195^ ^]^
Atg7 siRNA	Regulate autophagy and thus enhance chemotherapeutic apoptosis	DOX	Polymeric NPs	Pluronic P123‐PEI (600 kDa) (PP6)‐iRGD	Panc‐1 cells	Panc‐1 subcutaneous tumor model	Increase NPs penetration into tumors, improve therapeutic effect	^[^ ^185^ ^]^
miR‐9	Regulate autophagy and thus enhance chemotherapeutic apoptosis	DOX	Polymeric NPs	PL‐1 peptides	CFPAC‐1 cells, Panc‐1 cells, Capan‐1 cells	PDX tumor model	Improve the anticancer effect, induce apoptosis of PDAC tumors	^[^ ^186^ ^]^
sFlt‐1 pDNA	Suppress tumor angiogenesis and growth	–	Polymeric micelles	PEG‐pLys	BxPC3 cells	BxPC3 subcutaneous tumor model	Decrease vascular density, suppress tumor growth	^[^ ^189^ ^]^
VEGF siRNA	Suppress tumor angiogenesis and growth	–	Calcium phosphate NPs	PEG‐charge conversional polymer (CCP)	BxPC3 cells	BxPC3 subcutaneous tumor model	Enhance siRNA accumulation, silence VEGF gene (≈68%) in the tumor, suppress tumor growth	^[^ ^190^ ^]^

#### 3.3.1 Targeting Mutant KRAS

**Figure 9:**
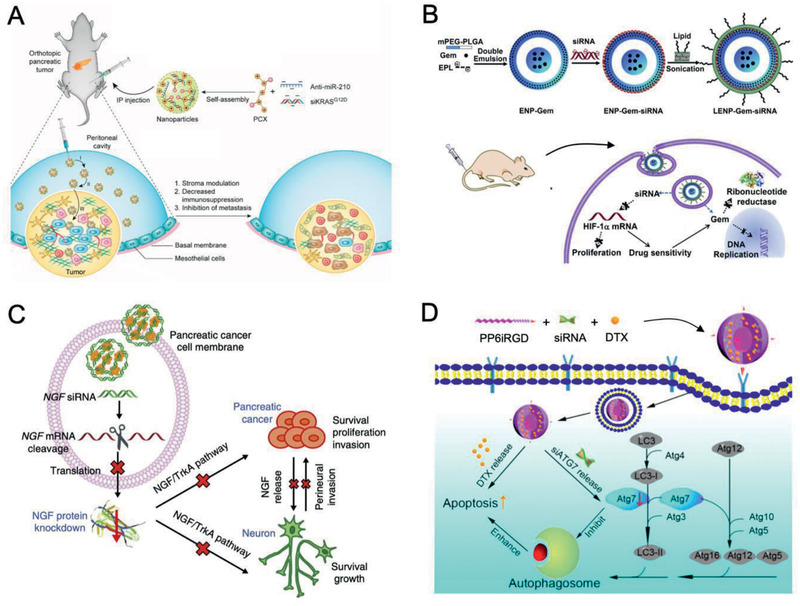
PDAC‐tailored nanomaterials for gene therapy. A) Scheme of PCX/(siKRAS + miR‐210) NPs for stromal modulation and metastatic PDAC therapy. Reproduced with permission.^[^ ^178^ ^]^ Copyright 2020, American Chemical Society. B) Schematic diagram of LENPs for codelivery of si‐HIF1a and GEM for synergistic antitumor therapy. Reproduced with permission.^[^ ^181^ ^]^ Copyright 2015, Elsevier. C) Schematic representation of GNC‐siRNA nanocomplexes for NGF silencing and PDAC therapy. Reproduced with permission.^[^ ^183^ ^]^ Copyright 2017, Springer Nature. D) Schematic illustration of PP6iRGD/DTX/siAtg7 micelles for improved DTX therapeutic outcomes. Reproduced with permission.^[^ ^185^ ^]^ Copyright 2019, American Chemical Society.

As a hallmark of PDAC, KRAS mutation is found in over 90% of PDAC cases and results in permanent activation of the KARS protein, which subsequently promotes the proliferation, invasion, transformation, and survival of the cells. Thus, its presence is closely correlated with the invasiveness of PDAC and the short survival of patients. Therefore, silencing mutant KRAS via siRNA drugs is a considerable approach with huge potential to treat PDAC, in which unfortunately, many attempts have been unsuccessful in the clinic. Kamerkar et al. engineered exosomes derived from normal fibroblast‐like mesenchymal cells to carry siRNA or shRNA specifically targeting oncogenic KRASG12D.^[^ ^177^ ^]^ And it has recently entered a phase I clinical trial of patients with metastatic PDAC (NCT03608631). In addition, Pei et al. constructed siRNA‐loaded apolipoprotein E3‐coated calcium phosphate (LCP) biomimetic NPs (siKRAS‐LCP‐ApoE3) to interfere with the oncogenic KRAS. After antifibrotic fraxinellone‐loaded NPs attenuated the dense stroma barrier, siKRAS‐LCP‐ApoE3 specifically targeted to ApoE3 receptors‐overexpressed KRAS mutant PDAC cells, silenced KRAS mutation and promoted ≈48.9% cell apoptosis. To further gain benefits in inhibiting tumor growth, the novel sequential targeting strategy was applied as it demonstrated significant inhibition in tumor growth (152.2 mm^3^ vs 1485 mm^3^ in PBS group), and concurrently elongated the median survivals of orthotropic PDAC animals (63 d vs 30.5 d in PBS group).^[^ ^154^ ^]^ Furthermore, Xie et al. fabricated cholesterol‐modified polymeric CXCR4 antagonist (PCX) NPs for codelivery of anti‐miR‐210 and siKRAS^G12D^. PCX/(siKRAS + miR‐210) NPs inactivated PSCs and promoted the infiltration of cytotoxic T cells, thus favorably reduced PDAC stroma and immunosuppression. The results obtained from orthotopic PDAC mice treated with PCX/(siKRAS + miR‐210) NPs demonstrated complete inhibition of liver metastasis, which improved animal survival by 50% in comparison with the control group (**Figure** **9**A).^[^ ^178^ ^]^ Alternatively, multifunctional graphene quantum dots (GQDs) with charged polyester coatings could load both siKRAS and DOX to synergistically inhibit the proliferation, cell migration, and invasion abilities of PDAC cells.^[^ ^179^ ^]^

#### 3.3.2 Targeting Hypoxia‐Inducible Protein

Due to the hypoxic PDAC microenvironment, densely distributed numerous signaling pathways are activated in tumor cells for tumor invasion, proliferation, and angiogenesis. Hypoxia‐inducible factor 1 (HIF1, composed of HIF1a and HIF1b) plays a critical role in hypoxia‐mediated apoptosis and simultaneously regulates tumor invasion, proliferation, angiogenesis and drug resistance.^[^ ^180^ ^]^ When the HIF1a siRNA (si‐HIF1a) and GEM were loaded into the biocompatible lipid‐polymer hybrid NPs via amphiphilic copolymer PEG‐PLGA, *ε*‐polylysine, and lecithin, the lipid bilayer shell exhibited a half lifetime longer than 3 h. As a result, si‐HIF1a was successfully protected against degradation in vivo. Hence, the carefully designed LENP‐Gem‐si‐HIF1a effectively suppressed innate immune activation and exhibited tumor metastasis inhibition in orthotopic PDAC models (Figure 9B).^[^ ^181^ ^]^ Other than the aforementioned protein complexes, the hypoxia‐inducible protein, endothelial PAS domain protein 1 (EPAS1) also contributes to tumor progression. Compared with the control group, EPAS1 siRNA NPs achieved twofold inhibition of tumor growth and a threefold decrease of microvessels formation in the xenograft PDAC models.^[^ ^182^ ^]^

In addition, increased neurite density is a frequent pathologic feature of PDAC, and more importantly, perineural invasion would result in undesirable severe neuropathic pain, high tumor recurrence and poor survival. Often, PDAC tissues actively induce the expression of neurotrophic factors, in particular, nerve growth factors (NGFs) to stimulate neurogenesis. Lei et al. hence developed Au NCs, which can efficiently deliver NGF siRNA (GNC‐siRNA) to silence NGF gene in PDAC. The delivered GNC‐siRNA nanocomplexes actively downregulated the NGF expression to block the activation of both PI3K‐Akt signaling pathway and RAS‐MAPK signaling pathway, thus superior suppression of the tumor growth in the subcutaneous model, orthotopic model and patient‐derived xenograft model were observed (Figure 9C).^[^ ^183^ ^]^

#### 3.3.3 Targeting Autophagy

Autophagy is essential for cell homeostasis and cellular metabolism, protecting healthy cells from malignant transformation.^[^ ^184^ ^]^ However, regarding to cancer cells including PDAC cells, the restoration of autophagy could inhibit ROS accumulation and DNA impairment, promoting the tumor progression, invasion, and metastasis.^[^ ^184^ ^]^ Therefore, autophagy inhibition via suppressing gene expression has been applied to significantly improve the effectiveness of PDAC therapy. Zhang et al. synthesized iRGD modified pluronic P123‐PEI (PP6iRGD) that can load autophagy‐related gene 7 specific siRNA (siAtg7) and DTX with the intention of tumor‐targeted delivery. As expected, the specific binding of PP6iRGD/DTX/siAtg7 micelles to integrins overexpressed on the endothelium of tumor vessels, promoted the silencing of Atg7 to inhibit autophagy induced by DTX, thus enhancing the DTX therapeutic outcomes in PDAC therapy (Figure 9D).^[^ ^185^ ^]^ Furthermore, Wu et al. assembled miR‐9 payloads with PL‐1 polypeptides for PL‐1‐targeted PDAC‐specific delivery. From their research, miR‐9 was discovered to give rise to the DOX sensitivity of PDAC cells via directly downregulating eIF5A2 expression to inhibit autophagy and cause apoptosis. The in vivo results demonstrated that PL‐1/miR‐9 NPs dramatically exacerbated the antitumor effects of DOX on PDAC xenografts, and implied that miR‐9/eIF5A2 might serve as a potential target for synergic therapy against PDAC.^[^ ^186^ ^]^

#### 3.3.4 Targeting TRAIL

Furthermore, as a member of the TNF superfamily, TRAIL has the unique property of killing tumor cells instead of normal cells; however, it has poor stability and short half‐life time in vivo.^[^ ^187^ ^]^ To overcome this dilemma, Na and coworkers paid close attention to the human mesenchymal stem cells (hMSCs), which own notable tumor‐homing capacity and great potential for gene delivery. The TRAIL gene (plasmid TRAIL, pTRAIL) was loaded with branched PEI, and the complex together with pheophorbide‐a (a photosensitizer) was then entrapped into hMSCs via photochemical internalization (PCI). In addition, the TRAIL gene transfection efficiency was enhanced by the employment of PCI. Hence, the TRAIL expressed from hMSCs and tumor‐homing ability of hMSC accelerated PDAC cell apoptosis. Consequently, the PCI‐pTRAIL/bPEI@hMSCs‐mediated therapy demonstrated a remarkable therapeutic effect in the Miapaca‐2 xenograft mouse model.^[^ ^188^ ^]^

#### 3.3.5 Targeting Angiogenesis

Besides, antiangiogenic gene therapy is another promising strategy, wherein tumor growth can be inhibited by destructing the neo‐vasculature formation. Vascular endothelium growth factor (VEGF) as a typical proangiogenic factor that promotes endothelial proliferation and migration. Kataoka and coworkers applied poly(ethylene glycol)‐poly(l‐lysine) (PEG‐pLys) block copolymers to develop cross‐linked polyplex micelles and deliver plasmid DNA (pDNA) encoding the soluble form of VEGF receptor‐1 (sFlt‐1). The polyplex micelles significantly decreased vascular density inside the tumor and inhibited the tumor growth.^[^ ^189^ ^]^ Furthermore, they prepared polymer/calcium phosphate (CaP)/VEGF siRNA hybrid NPs. The endosomal acidic condition of tumor cells can break the equilibrium between CaP complex and calcium ions and thus triggered the release of entrapped siRNA, which then significantly silenced the VEGF genes (68%) in the PDAC models.^[^ ^190^ ^]^

#### 3.3.6 Targeting miR‐21

With the recent development of miRNA research related to PDAC, certain types of miRNAs are identified to be overexpressed and regulate the initiation, proliferation, and invasion of PDAC. Gilles et al. studied data from a set of 191 patients and found that high expression level of miR‐21‐5p, miR‐196b‐5p, let‐7i‐5p, and miR‐196a‐5p are strongly correlated to the low survival rate in PDAC. Owing to the unique expression level of miR‐21, their effort was focused development of anti‐miR‐21 RNA loaded tumor‐penetrating nanocomplexes (TPN‐21) using N‐terminal fatty acid groups and cell penetrating peptide iRGD. Analysis of avatar results from patient‐derived‐organoid (PDO) revealed that TPN‐21 strongly restrained PDAC growth and had a tumor inhibition rate of 45.8% compared with the control group.^[^ ^191^ ^]^

#### 3.3.7 Targeting *β*III‐Tubulin

High *β*III‐tubulin (encoded by the TUBB3 gene) expression in human pancreatic tumors is closely associated with the chemoresistance and poor patient survival. As reported in multiple studies, sustained suppression of *β*III‐tubulin via shRNA can reduce pancreatic tumor growth and metastases significantly.^[^ ^192^ ^]^ Teo et al. synthesized star polymers with cationic poly(dimethylaminoethyl methacrylate) (PDMAEMA) side‐arms and poly[oligo(ethylene glycol) methyl ether methacrylate] (POEGMA) for siRNA delivery. By regulating the length of PDMAEMA side‐arms and the amount of POEGMA, star‐POEGMA polymers could readily self‐assemble with TUBB3/*β*III‐tubulin siRNA for safe and efficient siRNA delivery. Thus, high accumulation of the assembled star‐POEGMA‐siRNA was achieved and consequently silenced the *β*III‐tubulin expression up to 80% at both gene and protein levels in orthotopic pancreatic tumors.^[^ ^193^ ^]^

#### 3.3.8 Targeting Ubiquitin Ligase ITCH

In order to amplify the efficacy of conventional chemotherapeutic drugs, nanoscale RNAi targeting strategy is implemented to immensely improve the sensitivity and selectivity of the drugs. For example, p73 is a member of the p53 transcription factor family, whose upregulation sensitizes cancer cells to chemotherapy.^[^ ^194^ ^]^ In fact, the upregulation of the p53 is initiated by reducing the expression of the ubiquitin ligase ITCH, which regulates the p73 levels by ubiquitination and subsequent degradation via the proteasome. Likewise, p73 can be upregulated by reducing the expression of ITCH. de la Fuente et al. loaded antiubiquitin ligase ITCH siRNA and shRNA into generation 3 polypropylenimine dendrimer (DAB‐Am16). The combined usage of anti‐ITCH RNAi with GEM resulted in robust suppression in pancreatic tumor growth for 17 d even at suboptimal GEM exposure. It demonstrated that downregulation of ITCH by DAB‐Am16/shRNA sharply sensitizes PDAC cells to GEM and imparts the huge progress in PDAC therapy.^[^ ^195^ ^]^

In conclusion, the tailor‐made multifunctional nanomaterials for PDAC gene therapy is certainly promising as it prevents the gene invalidation, and increases the precise gene delivery to the site of the tumor. The approved ONPATTRO opens a new era of Nucleic Acids Nanomedicine, and nanotechnology‐enabled gene therapy would likely follow this success to be a life‐saving straw of PDAC patients.

### 3.4 PDAC‐Tailored Nanomaterials for Immunotherapy

Novel immunotherapeutic agents targeting cytotoxic T lymphocyte‐associated protein‐4 (CTLA‐4), and programmed cell death 1/programmed cell death 1 ligand 1 (PD‐1/PD‐L1) are revolutionizing the cancer therapy. However, the majority of PDAC excluding mismatch repair deficiencies are regarded to be resistant or immune‐quiescent.^[^ ^196^ ^]^ The results of a phase II trial of single agent Ipilimumab (anti‐CTLA‐4) against locally advanced or metastatic PDAC,^[^ ^197^ ^]^ and a phase I trial of mesothelin‐specific CAR‐T cells (CARTmeso cells) against chemotherapy‐refractory metastatic PDAC^[^ ^198^ ^]^ exhibited to be disappointing.

Researchers are making intensified efforts by using nanotechnologies to conquer the highly immunosuppressive PDAC microenvironment and broaden the impact of immunotherapy in PDAC. For instance, Huang and coworkers constructed liposome‐protamine‐DNA with plasmids encoding small trapping proteins targeting CXCL12 and PD‐L1 (termed pCombo trap NPs). The CXCL12 trap has the function of promoting the T cell penetration into tumor sites and PD‐L1 trap further accelerates T cell infiltration to kill PDAC cells. Thus, double‐step usage of pCombo trap NPs maximized their potential in significantly shrunk tumor volume, prolonged survival, and reduced metastasis of PDAC.^[^ ^165^ ^]^ Moreover, they have loaded the trap genes (IL‐10 trap and CXCL12 trap) to LPD NPs, which extensively decreased the immunosuppressive cells and activated immunosuppressive tolerogenic dendritic cells. As identified from the preclinical allograft PDAC models, LPD NPs effectively inhibited tumor growth by 20% after the treatments.^[^ ^162^ ^]^ In addition, Xie et al. loaded glucose oxidase (GOx) using CCM‐coated MSNs for combining the starvation therapy and immunotherapy. The CMSNs‐GOx has the capability of generating toxic H_2_O_2_ through GOx‐catalyzed glucose decomposition while amplifying the PD‐1 immune checkpoint blockade effect.^[^ ^199^ ^]^

Indoleamine 2,3‐dioxygenase (IDO), a metabolic enzyme, depletes the l‐tryptophan (Trp) present in both tumor cells and innate immune cells, which creates an immunosuppressive tumor microenvironment. Nevertheless, these immunosuppressive effects could be rescued by small molecule inhibitors of IDO, such as indoximod (IND). Hu et al. constructed hyaluronidase coated cationic albumin NPs (HNPs) to concurrently load IND as an IDO inhibitor, 1‐methyl‐tryptophan as an immune adjuvant, and celastrol for PDAC chemoimmunotherapy. The hyaluronidase‐triggered size‐reduction and CD44 receptor‐mediated endocytosis not only facilitated the accumulation but also promoted deep penetration into tumor tissues. The selected combination of IDO inhibition and chemotherapy ideally reversed the immunosuppressive tumor microenvironment. As such, enhancement in antitumor efficacy was evident from the in vivo studies of both xenograft and orthotopic pancreatic tumor models.^[^ ^200^ ^]^ By manipulating the MSNs, Lu et al. have shown delivery of the lipid‐conjugated IND prodrug (IND‐PL) and oxaliplatin (OX) for synergistically interfering immunosuppressive IDO pathway and inducing immunogenic cell death (ICD). Delicately designed OX/IND‐MSNs succeeded in enhancing the recruitment of CD8^+^ T cells along with the downregulation of Foxp3^+^ T cells and achieved significant tumor reduction as well as survival benefit.^[^ ^201^ ^]^

Notably, the immune cell, TAMs are prominent in highly immunosuppressive microenvironment of PDAC. In fact, M2 TAMs modify the tumor microenvironments and promote cancer growth via growth factors and cytokines.^[^ ^202^ ^]^ In such circumstances, reprogramming them towards the predominant antitumor M1 phenotype might hold great promise for effective cancer therapy. Thereby, Su et al. synthesized HA‐PEI/HA‐PEG self‐assembled NPs to deliver miR‐155/miR‐125b‐2 expressing plasmid DNA and develop miR‐155/miR‐125b‐2 modified tumor‐derived exosomes. These exosomes led to macrophages reprogramming to make M2 phenotypes macrophage rehabilitate to the M1 phenotype.^[^ ^203^ ^]^ He group recently developed M2 TAMs targeting micelles for codelivery of NVP‐BEZ 235 (PI3K‐*γ* inhibitor) and CSF‐1R‐siRNA. The synergetic inhibition of PI3K‐*γ* and CSF‐1R could remodel the tumor immune microenvironment and activate antitumor immune responses, which is very appealing for effective PDAC treatment.^[^ ^204^ ^]^

Despite the immense potential and the incredibly fast enhancement in nanotechnology for accomplishing effective immunotherapy on tumors, it is clear that PDAC is uniquely characterized by multiple redundant barriers to immunotherapy. It might be effective to combine approaches with chemotherapy as a therapeutic backbone, many of which are currently in clinical trials. Moreover, it has been reported recently, that autophagy promotes immune evasion of pancreatic cancer by degrading MHC‐I.^[^ ^205^ ^]^ Thus, the combination of immunotherapy and autophagy inhibition might be a promising therapeutic strategy against PDAC. Indeed, continuous efforts of the researchers are much in need to bring promising nanotechnology‐enabled immunotherapy to great success, and we anticipate that immunotherapy will become a key modality of future PDAC therapies.

### 3.5 PDAC‐Tailored Nanomaterials for Radiotherapy

Radiotherapy is an effective approach for clinical cancer therapy, during which ionizing radiation is introduced to generate substantial ROS in tumor tissues. The elevated ROS levels directly damage the DNA, lipids, and proteins, thereby causing cell death.^[^ ^206^ ^]^ Radiotherapy and chemoradiotherapy are sometimes used for PDAC in the resectable and adjuvant settings. The major goals of radiotherapy in these settings are to increase the likelihood of a margin‐negative resection, enhance local control and prevent disease progression. And radiotherapy is also widely used to palliate pain and bleeding or relieve obstructive symptoms in patients with PDAC that progressed or recurred locally.^[^ ^7^ ^]^

Although the tumor control rate improves with an increasing dose of ionizing radiation in radiotherapy, the high doses of ionizing radiations often result in severe damages to surrounding normal tissues. Therefore, radiosensitizers that absorb radiation rays have been widely exploited to improve therapy efficacy and meanwhile minimize the adverse effects on nearby tissues. Metformin,^[^ ^207^ ^]^ tolfenamic acid,^[^ ^208^ ^]^ CPA,^[^ ^209^ ^]^ nimotuzumab,^[^ ^210^ ^]^ selective checkpoint kinase 1 (Chk1) inhibitor MK8776,^[^ ^211^ ^]^ and WEE1 inhibitor AZD1775 ^[^ ^212^ ^]^ could sensitize PDAC cells to radiotherapy. For example, TA can downregulate antiapoptotic protein survivin, which is related to the resistance of radiation therapy, thus enhancing apoptosis and inhibiting cell proliferation.^[^ ^208^ ^]^ In addition, CPA is a potent SMO inhibitor, which can not only deplete CSCs and disrupt stroma, but also enhance the response of PDAC cells to ionizing radiation. Zhao et al. encapsulated CPA using block copolymers that were synthesized based on poly[oligo(ethylene glycol) monomethyl ether methacrylate]_31_‐block‐poly(2‐hydroxyethyl methacrylate)_26_ and hydrophobic oligo(*ε*‐caprolactone). The core‐cross‐linked polymeric micelles (M‐CPA) enhanced the radiation response, which increased 1.8 ± 0.2 fold, 1.5 ± 0.2 fold, and 1.5 ± 0.2 fold in Miapaca‐2 cells, L3.6pl cells and human pancreatic stellate cells (hPSCs), respectively. The results exhibited that combined M‐CPA and radiotherapy was effective for both PDAC cells and stellate cells.^[^ ^209^ ^]^

Moreover, inorganic metal nanomaterials containing high‐Z elements, such as Au, Bi, Pt, tantalum (Ta), with high X‐ray attenuation coefficients can serve as potential nanoscale radiosensitizers to enhance radiotherapy.^[^ ^78^ ^]^ These metal nanomaterials concentrate X‐ray energy within tumors to enhance DNA damage, through various physical processes including Compton scattering, Rayleigh scattering, and photoelectric effect.^[^ ^213^ ^]^ Au NPs have obtained extensive attention because of good biocompatibility and feasible chemical modification. Yoshida et al. prepared molecularly imprinted microgels (Au‐MIP microgels) by a one‐pot seeded precipitation polymerization method with Au NPs (24 nm), N‐isopropylacrylamide (NIPAm), and N,N′methylenebis(acrylamide) (MBAA) as seeds, main monomers and cross‐linking agents, respectively. The pancreatic tumor growth was markedly suppressed by Au‐MIP microgels treatment upon X‐ray irradiation.^[^ ^214^ ^]^ Furthermore, cerium oxide NPs (CONPs) with specific valence state (Ce^3+^ vs Ce^4+^) and oxygen defects possess both superoxide dismutase (SOD)‐like activity (scavenge superoxide radicals) and catalase‐like activity (scavenge H_2_O_2_) to modulate auto‐regenerative redox status. Wason et al. found that radiotherapy increased the SOD‐like activity and decreased the catalase‐like activity of CONPs in an acidic environment, while showed no influence on both SOD‐like and catalase‐like activities of CONPs at neutral pH. By switching the oxidation states between Ce^4+^ and Ce^3+^, CONPs contributed to radical accumulation for tumor selective apoptosis and kept normal tissues from the toxic side‐effect of radiation. Taken together, these results demonstrated that CONPs can act as both promising radiosensitizers for tumors and protective agents for normal tissues for improving PDAC treatment.^[^ ^215^ ^]^

The recent revolution in targeted radiosensitizers may provide many opportunities to decrease the indiscriminate toxicity to normal tissue. And radiotherapy in combination with other therapeutic modalities might lead to higher activity and lowering toxicity, due to the reduced dosages of both therapeutic agents and radiation. And the next step is to translate and test in well‐designed clinical trials.

### 3.6 PDAC‐Tailored Nanomaterials for Ablative Therapies

Numerous studies have explored laser and thermal local ablative therapies in various solid tumors, especially the unresectable locally advanced PDAC (stage III).^[^ ^216^ ^]^ In which, photodynamic therapy (PDT), photothermal therapy (PTT), magnetic hyperthermia (MH), radiofrequency ablation (RFA), ultrasound (US) therapy, and internal radionuclide therapy (IRT), are the typical therapies that locally destruct tumors to slow down disease progression.^[^ ^216^, ^217^ ^]^ The application of tailor‐made nanomaterials is promoted by the robust improvements in the effectiveness and safety of ablative therapies (**Table** **7**).

**Table 7:** Summary of studies on the PDAC‐tailored nanomaterials for ablative therapies

Ablative Therapy	Nanocarriers	Polymers/Macromolecules	Active Components	Targeting Ligands	Exogenous Radiation	Cell Lines	In vivo Model	Outcome	Ref
PDT	HSA NPs	HSA	Pheophorbide‐a (P@), GEM	–	670 nm laser, 10 mW cm^–2^	BxPC‐3 cells	BxPC‐3‐LN7 subcutaneous tumor model	Selective accumulation of NPs within the primary tumors and metastatic lymph nodes, enhance therapeutic effect toward cancer with lymphatic metastases	^[^ ^224^ ^]^
PDT	Polymeric NPs	PEI and PEG	Chlorin e6 (Ce6)	–	670 nm laser, 800 mW cm^–2^	AsPC‐1 cells, ABCG2‐overexpressing Miapaca‐2 cells	AsPC‐1 orthotopic/subcutaneous tumor model	Enhance intracellular Ce6 concentration and the PDT effect, reduce tumor volume	^[^ ^227^ ^]^
PTT	HSA‐paclitaxel@ liposomes	FAP‐*α* responsive cleavable amphiphilic peptide, DPPC	IR‐780 iodide	–	808 nm laser, 0.8 W cm^–2^	Panc‐02 cells, NIH3T3 cells	Panc‐02/NIH3T3 subcutaneous tumor model, Panc‐02 orthotopic tumor model	Promote the release of small sized HSA‐paclitaxel in deep tumor regions, enhance combined chemotherapy with PTT	^[^ ^229^ ^]^
PTT	Au NPs	Polymeric GEM‐ mono‐2‐methacyloyloxy ethyl succinate prodrug	Au NPs, GEM	–	640 nm laser, 1.4 W cm^–2^	Miapaca‐2 cells	–	Enhance thermal effect, synergistic photochemotherapeutic activity and significant cytotoxicity	^[^ ^231^ ^]^
PTT	Au NRs	Erythrocyte membrane	Au NRs, CPA	–	808 nm laser, 0.75 W cm^–2^	Capan‐2 cells	Capan‐2 subcutaneous tumor model	Significant shrinkage of Capan‐2 tumor xenografts	^[^ ^232^ ^]^
PTT	MSNs@Au nanoshell	PEG	Au nanoshell	Anti‐uPAR antibody	808 nm laser, 2 W cm^–2^	Panc‐1 cells, SW1990 cells	SW1990 orthotopic tumor model	Eradicate tumor cells, achieve tumor metastasis inhibition and cancer immunotherapy	^[^ ^233^ ^]^
PTT	Rod MSNs@Au nanoshell	Tf‐PEG	Au nanoshell, GEM	Tf	808 nm laser, 0.5 W cm^–2^	Miapaca‐2 cells	Miapaca‐2 subcutaneous tumor model	Improve GEM penetration and accumulation in tumor tissues	^[^ ^234^ ^]^
PTT	GQDs	Cationic polylactides with pendant tertiary amine groups	GQDs, DOX, siKRAS	–	650 nm laser, 0.2 W cm^–2^	Miapaca‐2 cells	–	Keep stable in physiologically mimicking media, promote KRAS downregulation activity, enhance bioactivity inhibition and anticancer activity	^[^ ^179^ ^]^
PTT	Multi‐walled carbon nanotubes (MWCNs)	PEG	MWCNs	–	808 nm laser, 2 W cm^–2^	Panc‐1 cells	–	Promote cellular damage in PDAC cells via the apoptotic pathway	^[^ ^236^ ^]^
PTT	Au‐GO	Zwitterionic chitosan	Au‐GO, DOX	–	808 nm laser, 3 W cm^–2^	Panc‐1 cells, Miapaca‐2 cells	Panc‐1 subcutaneous tumor model	Increase tumor uptake, enhance antitumor treatment, reduce toxicity	^[^ ^237^ ^]^
MH	Iron oxide NPs	Dimercapto‐succnic acid (DMSA)	Iron oxide NPs, GEM	Pseudopeptide NucAnt (N6L)	Alternating magnetic field (AMF to 43 °C)	BxPC3 cells, Panc‐1 cells	BxPC3 subcutaneous tumor model	Inhibit PDAC cell growth, induce PDAC cell death, reduce proliferation	^[^ ^240^ ^]^
MH	Iron oxide NPs	PLGA	Iron oxide NPs, 17‐ N‐allylamino‐17‐demethoxygeldanamycin (17AAG)	–	25 kOe AMF	Miapaca‐2 cells	–	Facilitate anti‐PDAC activity	^[^ ^242^ ^]^
MH	Iron oxide NPs, cetuximab‐conjugated, GEM‐containing magnetic albumin nanospheres (C225‐GEM/MANs)	BSA	Iron oxide NPs, GEM	Cetuximab	230 kHz AMF	AsPC‐1 cells, Miapaca‐2 cells	–	Increase apoptosis, enhance double‐targeted thermo‐chemotherapy against PDAC cells	^[^ ^241^ ^]^
MH	Iron/iron oxide NPs	3‐(3,4‐dihydroxyphenethylcarbamoyl) propanoic acid tetraethylene glycol ester	Iron/iron oxide NPs	–	145 kHz AMF	Panc‐02 cells	Panc‐02 disseminated peritoneal tumor model	Promote active delivery of NPs	^[^ ^244^ ^]^
RFA	Cetuximab‐conjugated Au NPs (10 nm), PAM4‐conjugated Au NPs (20 nm)	–	Au NPs	Cetuximab, PAM4 single‐chain IgG	600 W generator power RF field	Panc‐1 cells, Capan‐1 cells	Panc‐1 subcutaneous tumor model, Capan‐1 subcutaneous tumor model	Promote noninvasive induction of intracellular hyperthermia	^[^ ^253^ ^]^
RFA	Ni‐Au core‐shell nanowires (CSNWs)	–	Ni‐Au CSNWs	–	12–15 W, 900–950 MHz RF field	Panc‐1 cells	Panc‐1 subcutaneous tumor model	Induce PDAC cell death	^[^ ^254^ ^]^
US	Nab‐paclitaxel; BG8610, BG8214 microbubbles (MBs)	–	BG8610, BG8214 MBs, Nab‐paclitaxel	–	Frequency 1 MHz US	BxPC‐3 cells	BxPC‐3 subcutaneous tumor model	Decrease the tumor volume, increase therapeutic efficacy	^[^ ^259^ ^]^
US	Hollow MSNs‐l‐arginine‐CO_2_	–	CO_2_	–	1 W cm^–2^, frequency 1 MHz US	Panc‐1 cells	Panc‐1 subcutaneous tumor model	Inhibit tumor growth, reduce side effects	^[^ ^260^ ^]^
US	Cyclic decapeptide‐HMSNs‐l‐arginine	PEG	H_2_O_2_, l‐arginine as NO donors	Cyclic decapeptide CGLIIQKNEC	1 W cm^–2^, frequency 1 MHz US	Panc‐1 cells	Panc‐1 subcutaneous tumor model	Increase retention, inhibit tumor growth, prolong survival	^[^ ^262^ ^]^
SDT	Magnetic superparamagnetic iron oxide NPs and perfluorobutane (PFB) gas loaded MBs (MagMBs)	DBPC, DSPE‐PEG_2000_, DSPE‐PEG_2000_‐biotin	Rose Bengal, 5‐FU	Biotin	3.0 W cm^–2^, frequency 1 MHz US	BxPC‐3 cells, Miapaca‐2 cells, Panc‐1 cells, T110299 cells	BxPC‐3 orthotopic tumor model	Reduce tumor volume, promote sonodynamic/antimetabolite therapy	^[^ ^269^ ^]^
SDT	MagMBs	DBPC, DSPE‐PEG_2000_, DSPE‐PEG_2000_‐biotin	Rose Bengal, GEM	Biotin	Magnetic‐acoustic device (MAD): US: 1.17 MHz, Magnet: 0.2 T	BxPC‐3 cells, Miapaca‐2 cells	BxPC‐3 subcutaneous tumor model	Increase the therapeutic payload deposition ≈1.4 fold, decrease tumor volume 9% at 8 d after treatment	^[^ ^270^ ^]^
SDT	Fluorocarbon (FC)‐chain‐functionalized hollow MSNs (FHMSNs)	–	IR780	–	1 W cm^−2^, frequency 1 MHz US	Panc‐1 cells	Panc‐1 subcutaneous tumor model	Inhibit hypoxia‐induced resistance to SDT, promote killing and shrinkage of hypoxic PDAC	^[^ ^268^ ^]^

#### 3.6.1 PDT

As a noninvasive oncologic therapeutic modality, PDT induces cell death via ROS products, which are generated by the photochemical reactions between a photosensitizer (PS) and NIR laser.^[^ ^218^ ^]^ Regarding PS, the first generation (e.g., hematoporphyrin and photofrin, etc.), and second generation (e.g., modified porphyrins, pheophorbide‐a, phthalocyanines, and chlorins, etc.) with higher singlet oxygen quantum yield lay the foundation of the development of PS.^[^ ^219^ ^]^ Currently, PDT is well established in nonmelanoma skin cancers (basal cell carcinoma), all stages of head and neck cancer, as well as the nonneoplastic condition wet age‐related macular degeneration of the retina (AMD).^[^ ^220^ ^]^ Morevoer, there have been clinical trials conducted for locally advanced PDAC, where porfimer sodium,^[^ ^221^ ^]^ chlorin e6,^[^ ^222^ ^]^ and verterporfin (second‐generation)^[^ ^223^ ^]^ were applied as PSs for EUS‐guided PDT, separately. DeWitt et al. determined the safety and effect of EUS‐PDT on patients (*n* = 12; mean age, 67 ± 6 years; 8 male) with treatment‐naïve locally advanced PDAC (mean diameter, 45.2 ± 12.9 mm) in a phase I study. The change in pancreatic necrosis was assessed at 18 d after PDT via a CT scan. There were 6 of 12 patients (50%) showing an increase in the volume percentage of tumor necrosis as compared with baseline imaging. Notably, there was none of EUS or EUS‐PDT related adverse events occurred.^[^ ^221^ ^]^ This ensures the safety of PDT and its effectiveness for patients with locally advanced PDAC.

Many studies focus on the development of PS and their selectively targeting delivery to PDAC tissues. Yu et al. conjugated pheophorbide‐a to HSA and then encapsulated Gem to obtain P@‐Gem‐HSA‐NPs. Under the excitation of the 670 nm laser, P@‐Gem‐HSA‐NPs effectively produced ^1^O_2_ and optical fluorescence for imaging‐guided PDT. Gem and P@ as the constituents of NPs contributed to a remarkable synergistic therapeutic effect toward PDAC with lymphatic metastases.^[^ ^224^ ^]^ Furthermore, the two‐photon excited PDT was achieved by incorporating tetrasilylated porphyrin (PS1) into the walls of ethylene‐based periodic mesoporous organosilica NPs (PS1‐EPMOs), which can load GEM in the porous structure for the combined therapy. PS1‐EPMOs exacerbated the cytotoxic effects (≈20%) as compared to the group without irradiation (800 nm).^[^ ^225^ ^]^

However, the PDT efficacy is limited in cancer cells overexpressing ATP‐binding cassette protein ABCG2, because ABCG2 as they actively pump PS out of the cells.^[^ ^226^ ^]^ To address this problem, Na and coworkers developed cationic PS‐encapsulated polymeric NPs (PS‐pNPs), which were comprised of chlorin e6 (Ce6), PEI and PEG. PS‐pNPs augmented intracellular Ce6 concentration and singlet oxygen production in ABCG2‐overexpressing Miapaca‐2 cells. In addition, PDT mediated by PS‐pNPs sharply decreased the tumor volume in comparison with pure Ce6 treatment in both heterotopic and orthotopic PDAC models.^[^ ^227^ ^]^

Among variety of available therapies, PDT has received great attention as a promising and minimally invasive treatment for PDAC. Nevertheless, the essential component in PDT, PS, brings in undesirable adverse events such as skin photosensitivity. Therefore, additional studies are in demand to ensure the selective delivery of PS in tumors instead of other organs especially the skin.

#### 3.6.2 PTT

With employed NIR laser, PTT represents another potential therapeutic strategy to eradicate tumor cells by transforming light to sufficient heat via photothermal agents. To date, wide ranges of photothermal nanoagents composed of dye‐loaded nanomaterials, noble metal nanomaterials, nanocarbons, transition metal sulfide/oxides nanomaterials as well as organic molecules have been extensively explored.^[^ ^228^ ^]^ In particular, a type of Au nanoshells (termed as AuroShell particles; Nanospectra Biosciences Inc., Texas) was recently approved for a clinical trial in patients with refractory and/or recurrent tumors of the head and neck (NCT00848042). Although the current amount of clinical trials that reported about PTT on PDAC patients are limited, there are interesting discoveries about improving the PDAC treatment efficiency based on photothermal nanoagents.

Firstly, NIR dyes have been investigated to realize controlled and selective heating in the targeted tumor area. He group recently designed CAFs‐responsive thermosensitive liposomes to load IR‐780 (CAP‐ITSL) for PTT, which was constructed via the assembly of FAP‐*α* responsive cleavable amphiphilic peptides (CAP) with phospholipids (DPPC). Furthermore, HSA‐paclitaxel@CAP‐ITSL was formed through encapsulating HSA‐paclitaxel into CAP‐ITSL, which would release small sized HSA‐PTX in deep tumor regions after NIR laser irradiation. The hybrid HSA‐paclitaxel@CAP‐ITSL well combined PTT with chemotherapy, and demonstrated an excellent antitumor efficacy on both Panc‐02/NIH3T3 subcutaneous tumor models and Panc‐02 orthotopic tumor models.^[^ ^229^ ^]^

**Figure 10:**
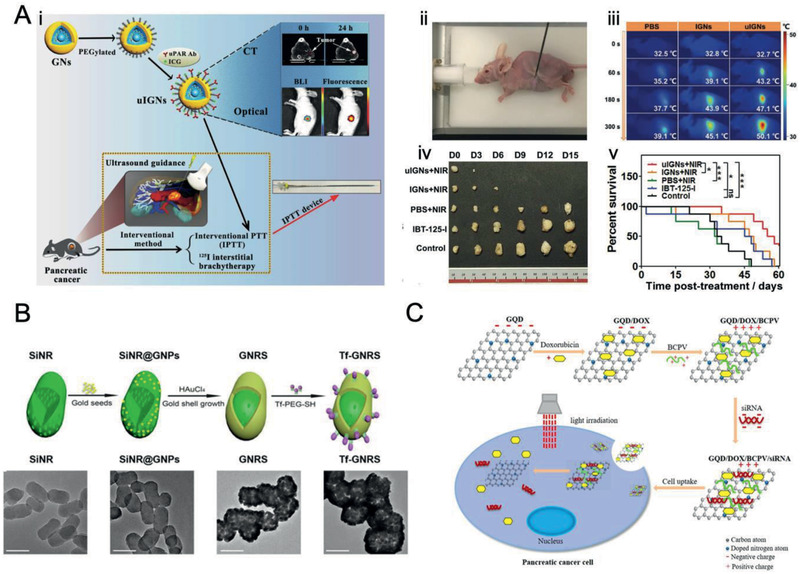
PDAC‐tailored nanomaterials for PTT. A) Schematic illustration of i) uIGN, ii) photoimage using a self‐developed IPTT device, iii) infrared thermal images for IPTT of PDAC, iv) photoimages of the resected tumors and v) percent survival in different treatment groups at 3 d interval. Reproduced with permission.^[^ ^233^ ^]^ Copyright 2017, Wiley‐VCH. B) Synthesis illustration of Tf‐GNRS and the corresponding TEM images. Scale bar, 100 nm. Reproduced with permission.^[^ ^234^ ^]^ Copyright 2017, American Chemical Society. C) Schematic illustration of GQD/DOX/BCPV/siRNA nanocomplexes for synergistic PTT and chemotherapy of PDAC. Reproduced with permission.^[^ ^179^ ^]^ Copyright 2019, American Chemical Society.

In addition, Au NPs can highly generate local hyperthermia upon NIR irradiation via the surface plasmon resonance (SPR) effect or electron vibrations.^[^ ^230^ ^]^ For example, polymer‐drug conjugates loaded Au NPs to possess synergistic photo‐chemotherapeutic activity against PDAC. The GEM‐Au hybrid NPs had a photothermal conversion efficiency as high as 63% and exhibited significant cytotoxicity against Miapaca‐2 cells with a red laser (640 nm).^[^ ^231^ ^]^ Jiang et al. synthesized Au NRs coated with RBC membranes, which exhibited excellent photothermal efficiency and induced dramatic shrinkage of Capan‐2 PDAC xenografts after irradiation with an 808 nm laser.^[^ ^232^ ^]^ In addition, Tian group synthesized a thin Au nanoshell with a spherical mesoporous silica nanocore (GNs) that were then conjugated by anti‐uPAR antibody, PEG, and ICG. The interventional PTT (IPTT) was mediated by using a NIR optical fiber through a percutaneous transhepatic cholangiography needle (18‐gauge) in an orthotopic PDAC xenograft model. The median survival rate of the uIGN group after IPTT was prolonged by 66.2% after NIR light irradiation (808 nm), compared to the control group (**Figure** **10**A).^[^ ^233^ ^]^ Furthermore, Nie group synthesized Tf‐modified Au nanoshell‐coated rod‐like MSNs (GNRS) with GEM payloads for PTT‐enhanced cascade‐targeting and thus improving PDAC therapy. Notably, accumulation of Au in tumor tissue increased from 186 ± 26 ng g^−1^ (GNRS group) to 206 ± 28 ng g^−1^ (Tf‐GNRS group) and 377 ± 40 ng g^−1^ (Tf‐GNRS/irradiation group), which was mediated in virtue of TfR targeting and photothermal effect‐enhanced permeability. Thereby, Tf‐GNRS have efficiently inhibited tumor regression by the synergistic effect of PTT induced cascade tumor targeting and chemosensitization (Figure 10B).^[^ ^234^ ^]^

Moreover, nanocarbons such as multi‐walled carbon nanotubes (MWNTs) can also be activated with NIR laser to elevate local temperature with nonlinear effects.^[^ ^235^ ^]^ Recently, Mocan et al. had reported MWCNTs‐PEG that promoted the mitochondrial membrane depolarization of Panc‐1 cells to induce cellular apoptosis under a laser excitation (808 nm).^[^ ^236^ ^]^ Moreover, Yang et al. synthesized nitrogen‐doped GQDs with superior drug loading efficiency. GQDs were modified by biodegradable charged polyester vectors (BCPVs) with pendant tertiary amine groups, which are available to encapsulate DOX and siKRAS. GQD/DOX/BCPV/siKRAS nanocomplexes generated heat with 650 nm laser irradiation to selectively trigger the simultaneous release of DOX and siKRAS, thereby significantly enhancing the anticancer effects (Figure 10C).^[^ ^179^ ^]^

Furthermore, integrating Au NPs and nanocarbons could remarkably enhance the heat conversion effect. Thapa et al. developed Au NPs‐decorated graphene oxide (Au‐GO) with the great NIR absorption ability and large surface areas, in which DOX were incorporated using zwitterionic chitosan (ZC) in a pH‐dependent manner. Au‐GO@ZC‐DOX nanovesicles exhibited pH‐triggered DOX release profiles, and strong photothermal effects upon NIR irradiation (808 nm), leading to potent anticancer effects and minimal toxicity in a Panc‐1 xenograft mouse model.^[^ ^237^ ^]^

Therefore, due to the high efficacy in tumor ablation with minimal damage to normal tissues, PTT is a promising strategy that might be translated into clinical practice in the near future, particularly for locally advanced PDAC.

#### 3.6.3 MH Therapy

MH therapy offers an alternative thermal ablation modality, which could be applied alone or as an adjunct to radiotherapy and/or chemotherapy to treat cancer.^[^ ^238^ ^]^ Some clinical trials of MH therapy have conducted in prostate cancer and brain cancer and multiple preliminary studies are revealing the great promise in PDAC treatment.^[^ ^239^ ^]^

Magnetic NPs (MNPs) like superparamagnetic iron oxide NPs can efficiently absorb and convert alternating magnetic fields (AMF) energy into heat. Sanhaji et al. conjugated MNPs with GEM and/or pseudopeptide NucAnt (N6L) for fostering the cellular uptake. The magnetic hyperthermia (up to 43 °C) led to severe cell death of BxPC‐3 cells (high sensitivity to GEM) and Panc‐1 cells (low sensitivity to GEM), and GEM combined therapy might well play a crucial role in optimizing local pancreatic tumor treatments.^[^ ^240^ ^]^ Besides, cetuximab‐conjugated and GEM‐loaded magnetic albumin nanospheres (C225‐GEM/MANs) were reported to specifically distinguish EGFR‐expressing PDAC cells and selectively generate concurrent hyperthermia against PDAC cells.^[^ ^241^ ^]^ Rochani et al. utilized PLGA to encapsulate iron oxide NPs and cytotoxic 17‐N‐allylamino‐17‐demethoxygeldanamycin (17AAG) as a Hsp90 inhibitor. The results exhibited that the combined Hsp90 inhibition and hyperthermia therapies effectively kill PDAC cells, and the hyperthermia under the magnetic field for 3 h resulted in ≈75% reduction in cell viability.^[^ ^242^ ^]^ In addition, core‐shell iron/iron oxide NPs are superior to iron oxide NPs to absorb the AMF, because the iron core contributes to a much stronger magnetization.^[^ ^243^ ^]^ Basel et al. loaded core‐shell iron/iron oxide NPs into RAW264.7 cells (monocyte/macrophage‐like cells, Mo/Ma) for tumor homing. MNPs‐loaded Mo/Ma specifically migrated into pancreatic tumors after intraperitoneal (i.p.) injection, and the AMF therapy led to a 31% increase in life expectancy post‐tumor insertion.^[^ ^244^ ^]^

Here, we envision that truly intelligent and real‐time diagnostic modalities would be a new direction in clinical practice, in which MNPs can achieve hyperthermia‐based treatment and drug delivery and also serve as appropriate clinical MR imaging contrast agents.

#### 3.6.4 RFA Therapy

RFA has a nonsurgical and minimally invasive nature and in the process of the treatment, it causes tumor necrosis by exerting high frequency alternating current to generate hyperthermia. It emerges as an effective local treatment for many unresectable and metastatic solid tumors. In fact, RFA therapy has been proved to be a safe treatment option especially for unresectable locally advanced PDAC under the optimal settings to prevent injury to the adjacent viscera.^[^ ^245^ ^]^ Multiple studies have reported that RFA therapy combined with simultaneous pancreatic resection or after curative resection can prolong the survival rate of some patients with liver metastasis.^[^ ^246^ ^]^

To further improve the efficacy of RF treatment and reduce the side effects on nearby tissues, multiple nanomaterials with high electrical conductivity including Au,^[^ ^247^ ^]^ nickel (Ni),^[^ ^248^ ^]^ iron,^[^ ^249^ ^]^ silver (Ag),^[^ ^250^ ^]^ QDs,^[^ ^251^ ^]^ and single‐walled carbon nanotubes (SWNTs) ^[^ ^252^ ^]^ have been introduced to intensify the heat inside target tissue. Glazer et al. prepared cetuximab‐conjugated Au NPs (10 nm Au) and PAM4 antibody‐conjugated Au NPs (20 nm Au) for RF field‐induced ablation of Panc‐1 and Capan‐1 PDAC xenografts, respectively. The results demonstrated that targeting antibody‐conjugated Au NPs significantly destructed tumors without causing injury to other organs after exposure to an RF field at 600 W as long as 6 weeks.^[^ ^253^ ^]^ Furthermore, Hopkins et al. synthesized Ni‐Au core‐shell nanowires (CSNWs) with the ability to generate heat and mediate heat induction. Ni‐Au CSNWs generated heat under RF irradiation (10 W) and led to severe necrosis, apoptosis, and fragmentation throughout the tumors.^[^ ^254^ ^]^

In order to avoid damage to important surrounding tissues and organs of patients, clinicians tend to perform RFA treatment only in the core zone of PDAC. Therefore, developing nanomaterials with higher electrical conductivity during the RF procedure or combining with other treatment modalities would be an effective way to expand the treatment area of the PDAC site and thus enhance the clinical benefits.

#### 3.6.5 US Therapy

US therapy is another locoregional therapy with noninvasive and high penetration depth that has been extensively applied for cancer therapy and diagnosis. Besides high‐intensity focused ultrasound (HIFU) therapy for local hyperthermia,^[^ ^255^ ^]^ nanotechnology‐enabled US‐triggered drug delivery,^[^ ^256^ ^]^ and sonodynamic therapy (SDT) have aroused increasing interests.^[^ ^257^ ^]^

The combined usage of US and MBs (1–10 µm) is an emerging approach for noninvasive and selective enhancement of drug delivery efficiency. The US activates gaseous MBs to enhance their permeability to endothelial barriers and cell membranes via the cavitation effect, thereby forming membrane pores and activating para‐ and trans‐cellular pathways. The sulfur hexafluoride MBs (SonoVue®)‐assisted US in combination with GEM has been proved to increase the median survival in inoperable PDAC patients (*n* = 10) from 8.9 months to 17.6 months (*p* = 0.011) without inducing any additional toxicity when compared to GEM alone.^[^ ^258^ ^]^ Moreover, Bressand et al. combined MBs‐assisted US with Nab‐paclitaxel to enhance intracellular uptake and significantly improve the therapeutic effectiveness of Nab‐paclitaxel in PDAC models.^[^ ^259^ ^]^

In regards to noninvasive and targeted therapy, the trigger‐responsive explosion (inertial cavitation) of the bubbles, e.g., CO_2_ bubbles, is a promising method to promote US therapy of PDAC. Shi group employed CO_2_ bubbles to construct an l‐arginine modified hollow MSNs system (HMSNs‐LA‐CO_2_) for US‐triggered tumor physical therapy. Upon exposure to US irradiation and/or endogenous acidic tumor environment, HMSNs‐LA‐CO_2_ responsively released a large amount of CO_2_ bubbles waves to cause cell necrosis via occluding blood supply within Panc‐1 tumor.^[^ ^260^ ^]^

In addition, US irradiation mediates the inertial cavitation‐mediated energy transformation from US mechanical energy to chemical energy (e.g., ROS, etc.), thus could be applied for US‐responsive drug delivery nanosystems. Considering the great potential of nitric oxide (NO) molecules in tumor therapy,^[^ ^261^ ^]^ Zhang et al. further employed HMSNs‐LA to construct an US‐responsive NO release system with the LA as a NO donor. US irradiation accelerated H_2_O_2_ decomposition to form highly active radicals, which rapidly oxidized HMSNs‐LA to generate more NO within tumor sites. These results demonstrated that HMSNs‐LA mediated Panc‐1 apoptosis by a DNA‐damage apoptotic pathway, and augmented the survival rate from 0% to 80% at 30 d after treatment in subcutaneously implanted Panc‐1 tumors.^[^ ^262^ ^]^

In comparison to photo‐inspired therapy, a new therapeutic modality, SDT has gained increasing attention with the numerous benefits and the opportunities for the noninvasive treatment of the deep tumors.^[^ ^263^ ^]^ SDT has adopted frequencies, which are in the nonthermal US frequency range (20 kHz‐3 MHz).^[^ ^264^ ^]^ Deriving from PDT, organic porphyrin ^[^ ^265^ ^]^ and their derivatives, such as hematoporphyrin,^[^ ^266^ ^]^ protoporphyrin (PpIX),^[^ ^267^ ^]^ are widely used as sonosensitizers in SDT at present. Chen et al. developed hollow MSNs to load IR780 sonosensitizers, and then functionalized them using fluorocarbon (FC)‐chain to deliver oxygen (a key substrate in SDT to generate ROS). By selectively releasing oxygen to diminish hypoxia‐induced resistance to SDT and produce more toxic ^1^O_2_ upon exposure to US irradiation, FHMSNs efficiently inhibited metastasis and shrunk hypoxic Panc‐1 pancreatic tumors.^[^ ^268^ ^]^ Sheng et al. further constructed magnetically responsive MBs (MagMBs) with an oxygen gas core and a phospholipid coating that were functionalized with superparamagnetic iron oxide NPs and biotinylated rose bengal (RB) sonosensitizer/5‐FU. Under the combined external magnetic and US fields, MagO_2_MB conjugates responsively retained at the tumor site and resulted in a 48.3% reduction in orthotopic BxPC‐3 pancreatic tumor volumes in comparison to the control group, whereas the US group resulted in a reduction of only 27.9%.^[^ ^269^ ^]^ They further applied a magnetic‐acoustic device (MAD) for the coalignment of the magnetic and ultrasound fields, which could simultaneously concentrate and activate the MBs at the target site. The in vivo results demonstrated that tumors treated with MagO_2_ MB‐RB‐Gem + MAD became 9% smaller at 8 d after treatment, while those treated with separate MagO_2_ MB‐RB‐Gem or devices alone were 45% and 112% larger, respectively.^[^ ^270^ ^]^ In addition, SDT can activate inorganic nanomaterials including nanoscale TiO_2_,^[^ ^271^ ^]^ Au,^[^ ^272^ ^]^ GO,^[^ ^273^ ^]^ MnWO*_x_*,^[^ ^274^ ^]^ and MOF‐derived mesoporous carbon nanostructures,^[^ ^275^ ^]^ to generate ROS for causing irreversible damage to tumor cells.

However, SDT‐related research is still in infancy, and nanotechnology‐enabled sonosensitizer is still very rare compared to PS in PDT due to the lack of deep understanding of the mechanism of SDT. Hence, we believe that the exploration of SDT mechanism and the synergistic treatment, such as combination with chemotherapies, gene therapy, radiotherapy as well as other modalities, could still be improved.

## 4 PDAC‐Tailored Theranostic Nanomaterials for Imaging‐Guided Therapy

Personalized medical intervention that can reflect patient characteristics and predict the therapy outcomes are needed for cancer patients. However, patients tend to receive follow‐up visits 4–6 weeks after the therapy to evaluate the therapeutic effect,^[^ ^276^ ^]^ which may influence the timely decision making for adjusting treatment plans. Imaging‐guided therapeutic strategies for PDAC that combine the diagnosis technology and treatment modality can provide detailed information of individuals during the therapeutic process, which can help to monitor the cancer prognoses and adjust the medical intervention.^[^ ^277^ ^]^ PDAC shows high risks of being recurred after curative surgery, and thus more precise and effective adjuvant therapy is strongly needed in PDAC therapy.^[^ ^278^ ^]^ Therefore, advanced imaging‐guided PDAC therapies have attracted broad attention in recent years.^[^ ^279^ ^]^ Herein, the research progresses made in PDAC‐tailored theranostic nanomaterials for imaging‐guided therapeutic strategies will be introduced (**Table** **8**).

**Table 8:** Summary of studies on the PDAC‐tailored theranostic nanomaterials for imaging‐guided therapy

Nanosystems	Targeting Ligands	Drugs	Imaging Components	Imaging	Source	Cell Lines	In vivo Model	Outcome	Ref
Au‐GO@ zwitterionic chitosan‐Cy5.5	–	Au‐GO, DOX	Au‐GO, Cy5.5	Optical imaging	No mentioned	Panc‐1 cells, Miapaca‐2 cells	Panc‐1 subcutaneous tumor model	Increase tumor uptake, enhance antitumor treatment, reduce toxicity	^[^ ^237^ ^]^
HSA‐GEM/IR780 nanocomplexes	–	GEM	IR780	Optical imaging	Maestro with 745 nm/785 nm filter setting	BxPC‐3 cells	BxPC‐3 subcutaneous tumor model	Enhance accumulation and long‐term retention, increase GEM concentration in the tumor tissue	^[^ ^60^ ^]^
PEG‐b[PLA‐*co*‐PMAC‐graft‐(IR820‐*co*‐GEM)] micelles	–	GEM	IR820	Optical imaging	Maestro system with 665 nm/785 nm filter setting	BxPC‐3 cells	BxPC‐3 subcutaneous tumor model	High cytotoxicity against BxPC‐3 cells, effective accumulation in tumor tissue, increase blood circulation time	^[^ ^54^ ^]^
MSNs‐GEM‐Au NC@BSA‐DOX	–	DOX	Au NC, hoechst	Optical imaging	Maestro with 645 nm and 461 nm	Miapaca‐2 cells	Miapaca‐2 subcutaneous tumor model	Enhance NPs accumulation and anticancer efficacy	^[^ ^285^ ^]^
HA functionalized Ag‐GQDs	HA	5‐FU	Ag‐GQD	Optical imaging	IVIS system with *λ*_ex_ 460 nm	Panc‐1 cells	Panc‐1 intraperitoneal tumor model	Fabricate targeted drug delivery, enhance anticancer efficacy	^[^ ^286^ ^]^
SWNT‐Cy7‐IGF1Ra	IGF1R antibody	–	SWNTs, Cy7	Optical imaging	IVIS system with *λ*_ex_ 745 nm and *λ*_em_ 800 nm	BxPC‐3 cells, Panc‐1 cells, ASPC‐1 cells, SW1990 cells	BxPC‐3 orthotopic tumor model	Induce tumor specific targeting, promote anticancer efficacy	^[^ ^56^ ^]^
GEM‐Gd supramolecular NPs		GEM	Gd	T1‐weighted MR imaging	7‐T MRI scanner	MDA‐MB‐231 cells	MDA‐MB‐231 subcutaneous tumor model	Prolong retention in tumor, increase antitumor activity, enhance diagnosis and therapy	^[^ ^288^ ^]^
CXCR4‐iron oxide NPs	CXCR4 monoclonal antibody	–	Iron oxide NPs (10 nm)	T2‐weighted MR imaging	1.5‐T MRI scanner	AsPC‐1 cells, BxPC‐3 cells, CFPAC‐1 cells, Panc‐1 cells	–	Enhance the cellular CXCR4 expression levels	^[^ ^71^ ^]^
Antimesothelin antibody‐conjugated PEGlyated liposomal DOX and iron oxide NPs	Antimesothelin antibody	DOX	Iron oxide NPs (5 nm)	T2‐weighted MR imaging	3‐T MRI scanner	Panc‐1 cells	Panc‐1 subcutaneous tumor model	Promote targeting distribution in the tumor xenograft, increase inhibition of tumor growth	^[^ ^290^ ^]^
Amino‐terminal fragment (ATF) peptide‐conjugated iron oxide NPs	ATF peptide	GEM	Iron oxide NPs (10 nm)	T2‐weighted MR imaging	3‐T MRI scanner, 4.7‐T animal MR scanner	Miapaca‐2 cells	Miapaca‐2 orthotopic tumor model	Enhance MR imaging, inhibit tumor cell growth, enhance therapeutic effect	^[^ ^291^ ^]^
Herceptin‐conjugated, iron oxide NPs and GEM‐loaded PLGA nanospheres (PGFIO)	Herceptin	GEM	Iron oxide NPs (13 nm)	T2‐weighted MR imaging	1.5‐T MRI scanner	Miapaca‐2 cells	Miapaca‐2 subcutaneous tumor model	Enhance MR imaging, induce tumor regression	^[^ ^289^ ^]^
Cetuximab‐conjugated, GEM‐loaded magnetic albumin nanospheres	Cetuximab	GEM	Iron oxide NPs (20 nm)	T2‐weighted MR imaging	7.0‐T animal MR scanner	AsPC‐1 cells, Miapaca‐2 cells	–	Increase apoptosis, enhance double‐targeted thermo‐chemotherapy against PDAC	^[^ ^241^ ^]^
GEM + Paclitaxel‐loaded PFP nanoemulsions	–	GEM, paclitaxel	PFP	US imaging	14‐MHz linear transducer	Miapaca‐2 cells	Miapaca‐2 orthotopic tumor model	Enhance killing of PDAC cells, control tumor growth	^[^ ^80^ ^]^
MSNs@Au nanoshell	Anti‐uPAR antibody	–	Au nanoshell, ICG	CT imaging, optical imaging	Xenogen IVIS system	Panc‐1 cells, SW1990 cells	SW1990 orthotopic tumor model	Eradicate tumor cells, promote cancer immunotherapy, inhibit tumor metastasis	^[^ ^233^ ^]^
Iron oxide NPs‐NHRITC/CO‐EPCAM	EPCAM antibody	–	Iron oxide NPs (20–30 nm), RITC	T2‐weighted MR imaging, optical imaging	7‐T MRI scanner	Panc‐1 cells	–	Monitor the interaction of NPs, promote multimodal imaging for diagnosis	^[^ ^95^ ^]^
Iron oxide NPs‐Cy5.5	Underglycosylated mucin 1 tumor antigen (uMUC1)	GEM	Iron oxide NPs, Cy5.5	T2‐weighted MR imaging, optical imaging	9.4‐T animal MRI, IVIS system with specific filters for the Cy5.5	–	KCM triple transgenic mice	Enhance uMUC1‐targeted imaging for predictive assessment of therapeutic response	^[^ ^94^ ^]^
GPC1‐GEM@HAuNC‐Cy7/Gd NPs	GPC1 antibody	GEM	Gd, Cy7	T1‐weighted MR imaging, optical imaging	3.0‐T MRI scanner, IVIS system	BxPC‐3 cells, Panc‐1 cells	BxPC‐3 orthotopic tumor model	Increase tumor inhibitory effect, promote early diagnosis, enhance tumor therapy	^[^ ^294^ ^]^
Iron oxide@Au NCs@erlotinib	Erlotinib	Erlotinib	Au NC	Optical imaging	–	Panc‐1 cells	–	Promote killing of EGFR overexpressed‐PDAC cells	^[^ ^295^ ^]^
IGF1‐ and NIR830‐conjugated iron oxide NPs	IGF1	DOX	Iron oxide NPs (10 nm), NIR 830	T2‐weighted MR imaging, optical imaging	4.7‐T animal MRI	Miapaca‐2 cells	PDX orthotopic tumor model	Detect NPs delivery and therapeutic responses, inhibit cell proliferation, induced apoptotic cancer cell death, enhance anticancer efficacy	^[^ ^296^ ^]^
Hydroxypropyl cellulose (HPC) grafted Iron oxide NPs @silica	–	GEM	Iron oxide NPs (7 nm), RITC, cyto780	T2‐weighted MR imaging, optical imaging	7‐T MRI scanner, IVIS system	Panc‐1 cells	Panc‐1 subcutaneous tumor model	Increase apoptotic cell death, enhance anticancer efficacy	^[^ ^297^ ^]^

### 4.1 Optical Imaging‐Guided Therapy

Optical imaging that utilizes NIR fluorescent light can provide real‐time information to the surgeons, thus can maximize tumor excision while minimizing collateral damage and the surgical time. Indeed, optical imaging‐guided therapy facilitates tumor‐targeted therapy with the gradual advances in the precision of surgery from tissue‐scale to molecule‐scale, and thus improves the surgical outcomes.^[^ ^49^ ^]^ Herein, we will introduce the optical imaging‐guided chemotherapy and surgical treatment for PDAC as well as the newly developed fluorescent metal NCs for optical imaging‐guided PDAC therapy.

The drug/NIR dye delivery systems are developed to monitor and enhance PDAC treatments. For example, Han et al. constructed an HSA‐GEM/IR780 nanocomplex through the conjugation of GEM to HSA via cathepsin B cleavable peptide GFLG, wherein IR780 was then encapsulated. The strong fluorescent signals in tumor tissues were observed under NIR excitation even at 72 h post‐injection of HSA‐GEM/IR780, and the tumor inhibition rate in HSA‐GEM/IR780 group (up to 63.0%) was much higher than that in free GEM group (13.8%). Therefore, HSA‐GEM/IR780 nanocomplexes could be employed as a theranostic agent for optical imaging‐guided PDAC therapy.^[^ ^60^ ^]^ Han et al. synthesized a polymeric prodrug PEG‐b‐[PLA‐*co*‐PMAC‐graft(IR820‐*co*‐GEM)] by ring‐opening polymerization and click reaction. The tumor signal intensity of prodrug NPs‐treated mice at 8 and 24 h post‐injection remained much higher than that of free IR820‐treated mice.^[^ ^54^ ^]^ Moreover, Thapa et al. loaded Cy5.5 into Au‐GO@ZC for optical imaging, wherein Cy5.5 fluorescence quenching was prevented by Au‐GO.^[^ ^237^ ^]^ Furthermore, Tian group coupled Cy7 and targeted IGF1R antibody with SWNTs. The SWNTs‐Cy7‐IGF1Ra emitted fluorescence under the laser irradiation to guide synergistic PTT caused by Cy7 and SWNTs in BxPC‐3 orthotopic tumor models.^[^ ^56^ ^]^

**Figure 11:**
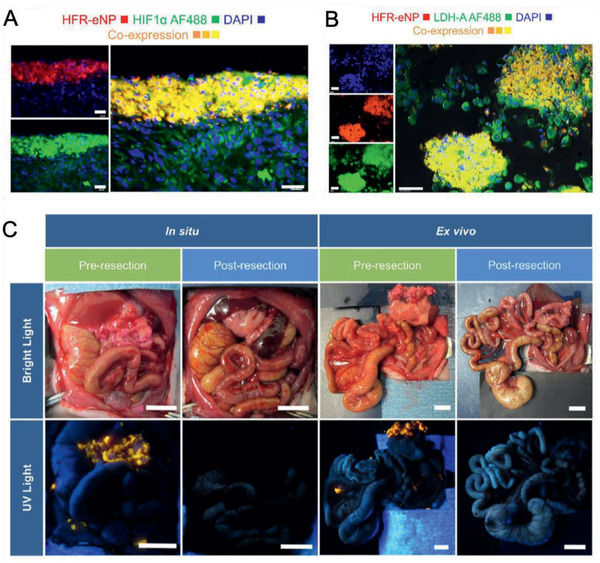
PDAC‐tailored nanomaterials for surgical resection. A) Colocalization of HRF‐eNPs and HIF1*α* (a marker for hypoxia). Scale bar, 40 µm. B) Colocalization of HFR‐eNPs and LDH‐A (a marker for tumor acidification). Scale bar, 40 µm. C) Bright light and corresponding UV light images before and after HFR‐eNPs guided cytoreductive surgery in a xenograft Panc‐1‐CSCs rat model. Scale bar, 1 cm. Reproduced with permission.^[^ ^283^ ^]^ Copyright 2017, American Chemical Society.

Apart from the optical imaging‐guided drug delivery, nanoscale NIR dyes can also guide fluorescence image‐guided surgery (FIGS) with a lower rate of false‐positive detection,^[^ ^280^ ^]^ because it is hard to differentiate pancreatic tumors from the healthy pancreas during surgical procedures and the unclear resection are highly associated with the reduced overall survival after the PDAC resection.^[^ ^281^ ^]^ Mohs groups reported on HA nanoformulation of ICG (termed NanoICG) for PDAC surgical resection. Surprisingly, NanoICG and ICG increased NIR signal in the tumor portion when compared with the healthy pancreas by 5.61‐ and 2.20‐fold in orthotopic pancreatic tumor models.^[^ ^282^ ^]^ In addition, Colby et al. reported fluorescent rhodamine‐labeled expansile nanoparticles (HFR‐eNPs) that localized to PDAC with high expression of lactate dehydrogenase‐A (LDH‐A). HFR‐eNPs achieved facile visualization of PDAC after i.p. injection using a hand‐held UV lamp. The significant reduction in fluorescent signals after resection revealed the valuable potential of HFR‐eNPs as intraoperative visual aids in surgical resections for submillimeter tumors (**Figure** **11**).^[^ ^283^ ^]^

In addition to the traditional fluorescent dyes, fluorescent metal NCs, such as Au, Ag, and Cu, are highly attractive fluorescent probes due to their size‐dependent fluorescence, excellent photostability and biocompatibility.^[^ ^284^ ^]^ Croissant et al. engineered MSNs‐Au NCs@BSA nanocarriers to encapsulate drug and hoechst. The in vivo imaging results demonstrated that both the red fluorescence from Au NCs and the blue fluorescence from Hoechst exhibited preferential pancreatic tumor bioaccumulation. It proved MSNs‐Au NCs@BSA can be applied as a multifunctional tumor theranostic nanoplatform.^[^ ^285^ ^]^ In addition, Joshi et al. explored a 5‐FU‐loaded Ag‐GQDs nanocomposite with the modification of carboxymethyl inulin (CMI) and HA. HA‐Ag‐GQDs‐5FU efficiently achieved targeted fluorescence bioimaging, significant tumor growth inhibition, and great biocompatibility.^[^ ^286^ ^]^

Optical imaging only requires simple and cost‐effective devices in comparison to MRI or CT, and can provide real‐time information to potentially help the decision making during the surgery of PDAC, which is vital for improving the surgical outcomes and the overall survival.^[^ ^50^, ^287^ ^]^ Therefore, developing nanosystems for optical imaging‐guided PDAC therapy show great potential to enhance the overall therapeutic outcomes.

### 4.2 MR Imaging‐Guided Therapy

The sensitive MR imaging is a powerful tool in the diagnosis of PDAC clinically as the codelivery of MR contrast agents and chemotherapeutic agents can achieve PDAC treatment under the guidance of MR imaging. Li et al. assembled Gd(III) and an active GEM metabolite into Gd supramolecular NPs (SNPs). SNPs enhanced T1‐weighted MR signal intensity in the tumor up to 1.47‐fold at 4 h post‐injection in comparison with the pre‐injection state. SNPs had a high drug loading ratio (55 wt%) and effectively inhibited MDA‐MB‐231 tumor growth, exhibiting the potential in MR imaging‐guided cancer therapy.^[^ ^288^ ^]^

To avoid the potential NSF toxicity of Gd‐based contrast agents, iron oxide NPs with lower risk of NSF were developed.^[^ ^68^, ^69^ ^]^ Wang et al. encapsulated iron oxide NPs with a diameter of ≈20 nm and GEM into the nanospheres to fabricate C225‐conjugated magnetic albumin nanospheres (C225‐GEM/MANs). Through the C225 and magnetic targeting strategies, C225‐GEM/MANs could efficiently distinguish EGFR‐expressing PDAC cells via T2‐weighted MR imaging and mediated thermo‐chemotherapy against PDAC cells.^[^ ^241^ ^]^ Jaidev et al. encapsulated iron oxide NPs and GEM in PLGA nanospheres (PGFIO) with functionalized with herceptin (an HER‐2 antibody). The multifunctional HER‐PGFIO nanospheres increased T2 contrast at tumor sites, and the chemotherapy‐MH group showed significant tumor regression (86 ± 3%) compared to the control group at the end of 30 days.^[^ ^289^ ^]^ In addition, Deng et al. prepared iron oxide NPs‐ and DOX‐loaded PEGlyated liposomes that were then conjugated by antimesothelin monoclonal antibody to target antimesothelin‐overexpressed PDAC cells. As shown in MR images, the change in tumor signal intensity induced of targeting liposomes (*Δ*SI = 145.98 ± 20.45) was much more remarkable than that by nontargeting liposomes (*Δ*SI = 75.69 ± 14.53) or free iron oxides NPs (*Δ*SI = 42.78 ± 22.12). A remarkable tumor growth inhibition of targeting liposomes demonstrated their fabulous ability for MR imaging‐monitored targeting therapy of human PDAC.^[^ ^290^ ^]^

Moreover, iron oxide NPs can be used as payload carriers for visual targeted delivery. For example, magnetic iron oxide NPs with a uniform core size of 10 nm can load GEM after surface modification by amino‐terminal fragment (ATF) peptides of uPA (ATF‐IONPs‐GEM). The efficient targeted delivery of ATF‐IONPs‐GEM into uPAR‐expressing PDAC cells and stromal cells, led to a 4.8‐fold T2‐weighted MR signal decrease in the tumor while much stronger tumor growth inhibition compared with nontargeted iron oxide NPs. The results confirmed that ATF‐IONPs‐GEM can real‐time monitor in vivo targeted delivery and achieve noninvasive MR imaging‐guided therapy.^[^ ^291^ ^]^

Notably, MR imaging‐guided PDAC treatment is becoming widespread in clinical practice for the diagnosis of PDAC. Furthermore, there are even clinically approved nanotechnology‐enabled contrast agents (e.g., iron oxide NPs). Therefore, iron oxide‐based nanomaterials exhibit great biocompatibility and potential for MR imaging‐guided PDAC therapy.

### 4.3 Multimodal Imaging‐Guided Therapy

Multifunctional nanomaterials that integrate multimodal imaging contrast agents and antitumor entities can overcome limitations of the single imaging modality and thus have drawn worldwide attention.^[^ ^292^ ^]^ Tian group applied ICG and uPAR antibody to modify Au nanoshells (called uIGNs). The uIGNs have exhibited excellent CT and NIR imaging owing to Au and ICG, respectively, and thus were used to monitor the therapeutic processes and tumor metastases (even less than 2 mm). More importantly, they have confirmed that uIGN successfully mediated IPTT for pancreatic tumor and prolonged survival period in mice. The minimally invasive uIGN‐mediated IPTT method is promising for clinical practice, particularly for high‐risk or advanced cancer patients.^[^ ^233^ ^]^

In addition, a series of targeting peptide binding NIR dyes cetuximab‐IRDye800,^[^ ^287^ ^]^ low‐density lipoprotein receptor (LDLR)‐Cy7,^[^ ^280^ ^]^ and EGF‐CF‐750^[^ ^293^ ^]^ can achieve specific and sensitive detection of orthotopic pancreatic tumors at deep depths via optical imaging and PA imaging, as well as guide surgical resection.

Furthermore, many researches detected in vivo targeted delivery of diagnostics nanomaterials in pancreatic tumors by combining optical imaging and MR imaging. Qiu et al. conjugated HA‐SH functionalized GEM@Au nanocages with Cy7, GPC1 antibody and Gd‐DOTA to fabricate multifunctional GPC1‐GEM@HAuNCs‐Cy7/Gd NPs. Both T1‐weighted MR signals and fluorescence signals have confirmed the arrival of GPC1‐GEM@HAuNCs‐Cy7/Gd NPs at the tumor site and peaked at 24 h post‐injection, demonstrating remarkable tumor inhibition effect in orthotopic pancreatic tumor models.^[^ ^294^ ^]^ Also, Nebu et al. synthesized erlotinib‐conjugated iron oxide‐Au NCs core‐shell nanocomposite for targeted optical and MR dual‐imaging and drug delivery.^[^ ^295^ ^]^ Zhou et al. applied amphiphilic polymer‐coated iron oxide NPs to encapsulate DOX and conjugate IGF1. Through targeted delivery of NIR830 dye‐labeled IGF1‐IONPs to IGF1R‐overexpressing pancreatic tumor cells and stromal cells, their fluorescence signals and T2‐weighted MR signals in the tumor were sharply higher than those of nontargeting iron oxide NPs at 24 h post‐injection. IGF1‐IONPs‐DOX inhibited cell proliferation and induced apoptotic cell death, leading to significant growth inhibition of orthotopic pancreatic PDX tumors.^[^ ^296^ ^]^ Furthermore, Kim et al. synthesized a temperature‐sensitive magnetic drug carrier to load iron oxide NPs (≈7 nm) and GEM. It achieved a faster release of GEM at 45 °C in comparison with that at 37 °C. And the GEM‐loaded magnetic carriers could significantly cause PDAC cell death under magnetic heating and achieve the visualization of tumors in the Panc‐1 xenografts model via both fluorescent and T2‐weighted MR imaging techniques.^[^ ^297^ ^]^

Although various novel nanomaterials with multi‐functions have been developed for imaging‐guided therapy of PDAC and showed impressive therapeutic efficiency, these systems are still in the preliminary animal studies. There are still many issues that need to be clearly verified to facilitate their clinical research, for example, biocompatibility, pharmacokinetics, large‐scale synthesis, and so on.

## 5 Conclusion

Despite the rigorous effort made in the development of functional nanomaterials, overall 5‐year survival in PDAC patients remains extremely low. The complex heterogeneous tumor microenvironment makes PDAC extremely refractory to the conventional therapeutic modalities. Therefore, there are various challenges that still need to be resolved in order to improve the diagnostic and therapeutic outcomes in PDAC.

Firstly, there are still difficulties in detecting PDAC at its early stages when treatment is most effective, resulting in a dismal prognosis of patients with locally advanced or metastatic disease. Therefore, there has always been an urgent need to increase the accuracy and sensitivity of laboratory tests and medical imaging for the early detection of PDAC lesions and the precise diagnosis of multi‐organ cancer metastasis to decrease the death rate. Herein, “off‐on” switchable nanoprobes assisted by responsive polymer,^[^ ^72^ ^]^ i‐motif DNA,^[^ ^74^ ^]^ or even chemical bond,^[^ ^73^ ^]^ provide new prospects for the early diagnosis of PDAC. Such “off‐on” switchable nanoprobes can selectively turn on the imaging signals triggered by the stimuli in tumor tissues while keeping the imaging signals powered off in normal tissues, thus exclusively achieving the selective amplification of imaging signals in tumors. To date, “off‐on” switchable nanotechnology has been proved to facilitate the highly sensitive diagnosis in animal models of hepatocellular carcinoma and is expected to be effective in sensitive early diagnosis of primary PDAC and synchronous metastasis.

Secondly, the limited therapeutic efficacy and severe systemic toxicity are a long‐standing problem of diagnostic and/or therapeutic nanomaterials. An individualized PDAC treatment by identifying and targeting specific altered genes or pathways would be an ideal approach. It is worth noting that PARP inhibitor olaparib (Lynparza) received FDA approval for germline BRCA‐mutation pancreatic cancer treatment in December 2019.^[^ ^298^ ^]^ Furthermore, the self‐renewable pancreatic CSCs with relatively higher expression level in ABC transporters, antiapoptotic proteins, and multidrug resistance genes, are crucially involved in multidrug resistance and metastasis of PDAC.^[^ ^299^ ^]^ Their biomarkers (e.g., CD133, CD24, CD44, CXCR4, EpCAM, Oct4, ABCB1, ABCG2, c‐Met, ALDH‐1, and nestin, etc.),^[^ ^300^ ^]^ multiple signaling pathways (e.g., Wnt/*β*‐catenin, SHH, Notch, and mTOR pathways),^[^ ^301^ ^]^ and unique metabolic suppression (e.g., oxidative phosphorylation (OXPHOS),^[^ ^302^ ^]^ glutamine^[^ ^303^ ^]^) could be considered to construct CSCs‐tailored nanomaterials for enhanced PDAC therapy. Regarding to the design and fabrication of targeting nanomaterials, the stimuli‐responsive “off‐on” switchable nanomaterials that can be selectively activated to be toxic in PDAC tumor tissues while remaining inactive in normal tissues are promising approaches. However, their scaling‐up and in‐process controls, as well as possible in vivo long‐term toxicity are still remaining to be solved.^[^ ^304^ ^]^

Thirdly, despite many positive effects that have been made on enhanced drug delivery, anti‐stromal therapy can be a double‐edged sword. The stroma ablation or CAFs depletion can promote the unchecked proliferation of tumor cells and heighten the risk of tumor dissemination. For example, the PDAC in the absence of epithelial‐derived SHH secretion presented few desmoplastic stroma, which were much more vascularized and proliferative than the control.^[^ ^149^, ^305^ ^]^ Researchers should particularly be focused on targeting both stromal and tumor compartments through the combination of antistroma therapy with chemotherapy, immunotherapy, or gene therapy. For example, since fewer CAFs in tumors were correlated with reduced survival, anti‐CTLA4 immunotherapy reversed disease acceleration and prolonged animal survival, which highlighted the potential efficacy of immunotherapy in combination therapies.^[^ ^150^ ^]^ Therefore, the finely tuned combination therapy for PDAC might bring tremendous therapeutic opportunities while decreasing the risk of tumor metastasis.

Lastly, clinic translation for various nanomaterials with great performances in animal models have been suspended due to the limited ability of animal models to recapitulate the stromal characteristics and intact immune system of human PDAC tumors. Thus, the patient‐derived xenograft model or genetically engineered mouse model (GEMM) is highly recommended to propel these nanomedicines from bench to bedside.

This review comprehensively highlights the versatile tailor‐made nanomaterials for enhanced diagnostics and treatment of PDAC. With a deeper understanding of the nano–bio interactions that occur in the unique PDAC microenvironment, it is increasingly important for material scientists, clinicians, pharmacists, and other researchers to integrate ideas into the development of novel tailor‐made nanomaterials both preclinically and clinically, especially in the areas of nanotechnology‐enabled biosensors, imaging, and therapeutic drug delivery. From the nanotechnological perspective, we expect multifunctional nanomaterials that focus on both killing tumor cells and modulating heterogeneous PDAC microenvironment would be extremely promising; and with their rapid development, their clinical translation is on the horizon. We hope this review can inspire the design and fabrication of PDAC‐tailored functional nanomaterials for early diagnosis and individualized treatment to raise the clinical benefits in PDAC patients.

## Conflict of Interest

The authors declare no conflict of interest.
